# Centenarians with proximal humeral fracture

**DOI:** 10.1186/s12877-025-06820-w

**Published:** 2025-12-02

**Authors:** Jeanette Koeppe, Julia Sußiek, J. Christoph Katthagen, Karen Fischhuber, Jan P. Happe, Janette Iking, Ursula Marschall, Andreas Faldum, Michael J. Raschke, Josef Stolberg-Stolberg

**Affiliations:** 1 Institute of Biostatistics and Clinical Research, University of Muenster, Schmeddingstrasse 56, Muenster, 48149 Germany; 2 Research group “Mathematical Surgery”, University Hospital Muenster, University of Muenster, Muenster, Germany; 3 Department of Trauma-, Hand- and Reconstructive Surgery, University Hospital Muenster, Albert-Schweitzer-Campus 1, Building W1, Muenster, 48149 Germany; 4 BARMER Institute for Health System Research, Lichtscheider Strasse 89 12, Wuppertal, 42285 Germany

**Keywords:** Proximal humeral fracture, Centenarians, Geriatric surgery, Level of care, Health service research

## Abstract

**Background:**

The demographic change in Germany leads to an increased number of centenarians. Within this population fragility fractures, such as the proximal humeral fracture (PHF), are not well investigated. This study aims to evaluate the epidemiology, treatment and outcome after a PHF in patients ≥ 100 years of age in Germany.

**Methods:**

Retrospective claims data of the BARMER health insurance were analyzed. All in- and outpatient cases of insurance holders ≥ 65 years from 01/2011 to 09/2022, with coded diagnosis of PHF were analyzed. The patients aged 65–99 years were used as a comparison group for the centenarians. Primary endpoints were overall survival, major adverse events (MAEs) and thrombo-embolic events.

**Results:**

In total, 100,482 patients with PHF were included in the study, with 230 aged 100 years and older. Centenarians with a PHF were more often female with several age-associated comorbidities, but less life-style associated risk factors. Centenarians were less often treated surgically. The outcome after PHF was worse with increasing age, 59.2% of the centenarians died one year after the PHF.

**Conclusion:**

The majority of centenarians sustaining a proximal humeral fracture was female. The most common treatment was the non-operative therapy. Nevertheless, also the operative therapy is associated with a low complication rate. As expected, the mortality rate is high, with almost 60% of deceased patients after one year. It remains unclear, if the mortality was related to proximal humeral fractures.

**Level of evidence:**

Level III, retrospective comparative study.

**Supplementary Information:**

The online version contains supplementary material available at 10.1186/s12877-025-06820-w.

## Introduction

The demographic change in Germany results in a rising number of older patients. Simultaneously, the amount of people 100 years of age and older increases drastically: In 2024 there are 1.808 centenarians per 100.000 habitants and it is assumed that the number will double in Europe until 2050 [1]. With increasing older patients suffering from musculoskeletal diseases, health care provision and medical decision making will be challenging particularly in centenarians [2]. While the hip fracture in the centenarian population has been investigated in several studies [3, 4], there is actually no literature available regarding proximal humeral fracture (PHF). It is the third most common fracture in older people with an incidence above 350 per 100.000 inhabitants [5, 6]. There remains a controversial debate regarding the treatment algorithm of proximal humeral fractures in the elderly population. In case of operative treatment, open reduction and internal fixation with a locking plate fixation is frequently used. However, in the last years the proportion of reverse total shoulder arthroplasty (RTSA) is increasing [7, 8]. There is contradictory evidence regarding the outcome comparing the operative with non-operative therapy. Recently Katthagen et al. demonstrated that the non-operative therapy comes along with a lower overall complication rate but with more major adverse events, such as thromboembolic incidents and a higher mortality [9]. On the other hand, Cheng et al. confirmed a similar overall complication rate for operative and non-operative treatment [10]. Therefore, the treatment choice mostly remains a decision based on patient’s individual factors.

With this study at hand, we therefore evaluated a large German insurance database regarding the following questions: What is the epidemiology of proximal humeral fractures in patients ≥ 100 years? How are centenarians with a proximal humeral fracture treated and what is the outcome?

## Materials and methods

### Data pool and patient cohort

The German remuneration system is characterized and regulated by mandatory coding instructions [7, 9, 11–13]. Encoding of diagnosis is based on the International Statistical Classification of Diseases, German Modification (ICD-10 GM). Procedures are encoded using German procedure classification (OPS).

For the study at hand, patient remuneration data (inpatient and outpatient data) of the BARMER health insurance were available. All older patients (age ≥ 65 years) with in- or outpatient coded proximal humeral fracture (ICD S42.2) between 01/2011 and 09/2022 were included to the study, with exclusion criteria as presented in supplementary figure S1. The first coded diagnosis of proximal humeral fracture was defined as the index event of the study. Patients were classified per age (65–69 years, 70–79 years, 80–89 years, 90–99 years, ≥ 100 years). Comorbidities and prior pharmaceutical treatment were detected within 24 months before proximal humeral fracture. Definitions for all variables and endpoints are presented in supplementary table S1. With a median follow-up time of 67.7 months, all patients were observed from date of proximal humeral fracture (inpatient: admission date; outpatient: date of imaging) to end of follow-up, defined by exit from the database, death or end of the study (31/12/2022). Hospital Frailty Risk Score was determined according to Gilbert et al. 2018 [14], but without modification to German ICD system.

### Primary and secondary endpoints

Primary endpoints were defined as overall survival (OS), major adverse events (MAE; defined as resuscitation, acute myocardial infarction, stroke, sepsis, acute renal failure, acute liver failure, acute respiratory distressed syndrome or death), thromboembolic events (TE; defined as deep vein thrombosis, pulmonary embolism, ischemic stroke or death) and surgical or injured-related complications (SCs; definition see suppl. Table S1). Secondary with osteoporosis-associated fractures (OAFs; including distal radius fractures, proximal femur fractures, fractures of the vertebrae, pelvic ring fractures), worsening of the level of care (LoC) and minor outpatient complications (MOCs) were defined as secondary endpoints.

### Missing values

Missing information about basic data, such as sex, date of birth or date of death were defined as exclusion criteria. Apart from this, no missing data occurred in the study, since all variables were defined by existing ICD or OPS codes. If no related code was found, the variable was set to zero.

### Statistical methods

**Fig. 1:**
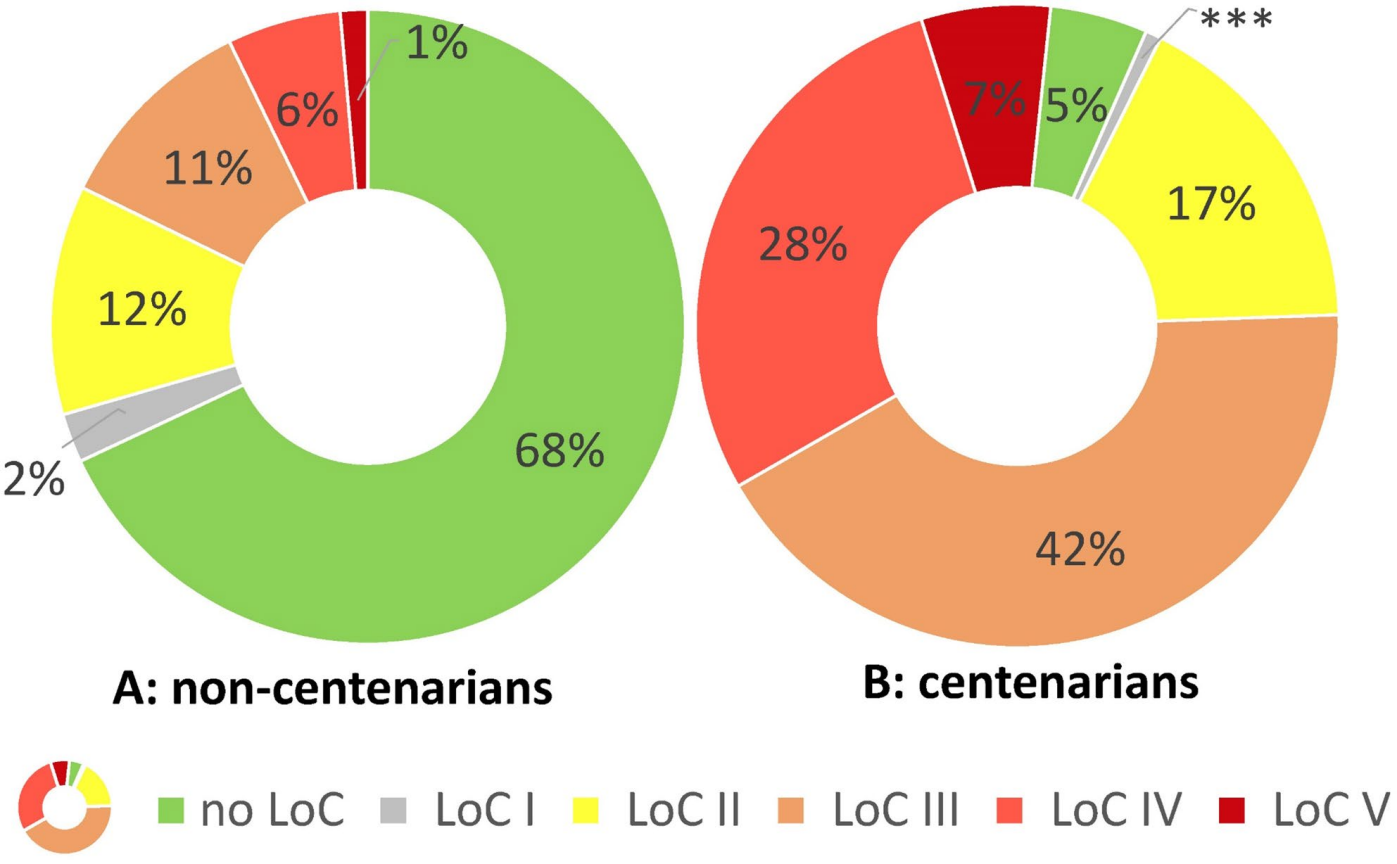
Distribution of level of care preexisting to proximal humeral fracture. Only patients treated from 2017 onwards were included (age < 100years: *N* = 53,812; age ≥ 100 years: *N* = 123). *** censored due data privacy protection

Patient characteristics, treatment and level of care preexisting to proximal humeral fracture were analyzed descriptively according to the age at proximal humeral fracture (grouped in centenarians/non-centenarians and/or in age-grouped defined above). For clarity of presentation, descriptive comparisons were made between patients aged ≥ 100 years and those aged < 100 years (Table 1; Fig. 1). This grouping was chosen to highlight centenarians as a unique subgroup of interest. Outcome analyses, however, were performed across 10-year age strata to ensure robust and consistent comparisons. To analyze the endpoints overall survival, major adverse events, thromboembolic events and worsening of the level of care (or death), the survival function was determined depending on age using Kaplan-Meier estimates. To analyze the influence of age on the outcomes, multivariable Cox proportional hazard models including sex, age and comorbidity profiles were used. Surgical treatment within 21 days was considered as time-dependent co-variable. For worsening of the level of care, only patients from 2017 to 2019 were included, since the definition of level of care in Germany was changed at 2017.

**Table 1:** Baseline characteristics. Patients were group by age at proximal humeral fracture (PHF). Simple-fracture Locked Plate Fixation – sLPF, Locked Plate Fixation multi-fragmented fractures – LPF, Reverse Total Shoulder Arthroplasty RTSA, Open Reduction and Internal Fixation – ORIF. *** censored due data privacy protection.

Parameter	Entire cohort	Non-centenarians (age < 100 years)	Centenarians (age ≥ 100 years)
Frequency – n (%)	100,482 (100.0%)	100,252 (99.8%)	230 (0.2%)
Female sex – n (%)	84,145 (83.7%)	83,941 (83.7%)	204 (88.7%)
Sector of diagnosis – n (%):			
inpatient	60,443 (60.2%)	60,280 (60.1%)	163 (71%)
outpatient	40,039 (39.9%)	39,972 (39.9%)	67 (29%)
Surgical treatment within 21 days after diagnosis – n (%):			
Total	45,892 (45.7%)	45,847 (45.7%)	45 (19.6%)
sLPF	*** (3.4%)	3,443 (3.4%)	< 5
LPF	21,977 (21.9%)	21,967 (21.9%)	10 (4.4%)
RTSA	9,433 (9.4%)	9,426 (9.4%)	7 (3.0%)
Other ORIF	17,459 (17.4%)	17,429 (17.4%)	30 (13.0%)
Osteoporosis – n (%)	35,090 (34.9%)	34,987 (34.9%)	103 (44.8%)
No. of prior osteoporosis-assoc. fractures within 5 years before PHF – n (%)			
0	77,660 (77.3%)	77,529 (77.3%)	131 (57.0%)
1	17,503 (17.4%)	17,429 (17.4%)	74 (32.2%)
2	4,267 (4.3%)	4,248 (4.2%)	19 (8.3%)
3	903 (0.9%)	897 (0.9%)	6 (2.6%)
4	143 (0.1%)	143 (0.1%)	0 (0.0%)
5	6 (0.0%)	6 (0.0%)	0 (0.0%)
Type of prior osteoporosis-assoc. fractures within 5 years before PHF – n (%)			
Distal radius	*** (2.1%)	2,055 (2.1%)	< 5
Proximal femur	10,635 (10.6%)	10,563 (10.5%)	72 (31.3%)
Vertebrae	10,639 (10.6%)	10,606 (10.6%)	33 (14.4%)
Pelvic ring fractures	3,966 (4.0%)	3,951 (3.9%)	15 (6.5%)
Living in nursing home prior to PHF – n (%)	8,209 (8.2%)	8,104 (8.1%)	105 (45.7%)
Hospital Frailty risk score – median (Q1,Q3)	8.9 (4.0, 17.3)	8.9 (4.0, 17.3)	18.1 (11.2, 26.9)
Cancer	27,599 (27.5%)	27,541 (27.5%)	58 (25.2%)
Diabetes mellitus – n (%)	30,499 (30.4%)	30,441 (30.4%)	58 (25.2%)
Dementia – n (%)	13,436 (13.4%)	13,354 (13.3%)	82 (35.7%)
Chronic polyarthritis – n (%)	6,449 (6.4%)	6,444 (6.4%)	5 (2.2%)
Omarthrosis – n (%)	2,904 (2.9%)	2,898 (2.9%)	6 (2.6%)
Frozen shoulder – n (%)	4,273 (4.3%)	4,268 (4.3%)	5 (2.2%)
Obesity – n (%)	20,254 (20.2%)	20,248 (20.2%)	6 (2.6%)
Nicotine abuse – n (%)	*** (6.5%)	6,534 (6.5%)	< 5
Alcohol abuse – n (%)	*** (4.9%)	4,910 (4.9%)	< 5
Parkinson disease – n (%)	*** (4.0%)	4,029 (4.0%)	< 5
Rotator cuff rupture – n (%)	2,594 (2.6%)	2,594 (2.6%)	0 (0.0%)
Atrial fibrillation/ flutter – n (%)	19,546 (19.5%)	19,493 (19.4%)	53 (23.0%)
Congestive heart failure – n (%)	24,694 (24.6%)	24,574 (24.5%)	120 (52.2%)
Coronary heart disease – n (%)	26,090 (26.0%)	26,005 (25.9%)	85 (36.7%)
Hypertension – n (%)	82,989 (82.6%)	82,792 (82.6%)	197 (85.7%)
Atherosclerosis – n (%)	16,644 (16.6%)	16,612 (16.6%)	32 (13.9%)
Chronic kidney disease – n (%)	25,011 (24.9%)	24,917 (24.9%)	94 (40.9%)
Previous stroke and other cerebrovascular disease – n (%)	27,247 (27.1%)	27,180 (27.1%)	67 (29.1%)
Previous medication:			
Any anticoagulant – n (%)	31,957 (31.8%)	31,884 (31.8%)	73 (31.7%)
Calcium/ Vitamin D – n (%)	7,178 (7.1%)	7,164 (7.2%)	14 (6.1%)
Bisphosphonates – n (%)	6,636 (6.6%)	6,630 (6.6%)	6 (2.6%)
Any anti-osteoporotic treatment – n (%)	12,278 (12.2%)	12,258 (12.2%)	20 (8.7%)

For all other endpoints, death was considered as a competing risk event and event rates were determined by cumulative incidence function using Aalen-Johansen estimates. Multivariable modelling was analogues done as described above but now using Fine and Gray models to estimate sub-distributional hazard ratios (HRs).

To account for possible differences in the treatment effect between age groups, all endpoints were also analyzed including an interaction term between treatment (surgical treatment within 21 days after proximal humeral fracture yes/no, time dependent) and age group.

Hazard ratios or sub-distributional HRs with 95% confidence interval (95%CI) were presented. Full model results were presented in supplementary table S5.

All analyses were fully explorative without adjustment for multiple comparison problem and all p-values were interpreted accordingly in the sense of hypothesis generation. For statistical analyses, SAS Enterprise Guide Version 8.3 Update 2 (SAS Institute Inc., Cary, NC, USA) and R (R version 4.2.0 (2022-04-22) R Foundation for statistical computing, Vienna, Austria) were used.

## Results

### Demographics and comorbidities

In total, 100,482 patients with proximal humeral fracture were included in the study, with 230 patients aged 100 years and older. Centenarians with a proximal humeral fracture were more often female (centenarians 88.7% vs. 83.7% in comparison group), were less often treated surgically (centenarians: 19.6% vs. non-centenarians 45.7%) and less often diagnosed in an outpatient setting (29.0% vs. 39.9%; see Table 1 and supplementary figure S2). Even though centenarians were more affected from age-associated comorbidities – e.g. dementia (35.7% vs. 13.3%), chronic heart failure (52.2% vs. 24.5%) or chronic kidney disease (40.9% vs. 24.9%) – life-style associated risk factors were less common in those patients with an age over 100. In detail, alcohol abuse (centenarians < 2% vs. 4.9%), nicotine abuse (centenarians < 2% vs. 6.5%) and obesity (2.6% vs. 20.2%), were less often coded during the evaluated pre-phase of 24 months before proximal humeral fracture (see Table 1). In addition, centenarians had a much higher Hospital Frailty Risk Score (centenarians: median 18.1 vs. median 8.9 in non-centenarians) and the distribution of the pre-existing level of care differed with a clear shift to higher level of care (see Fig. 1).

### Association of age and the outcome after proximal humeral fracture

**Fig. 2:**
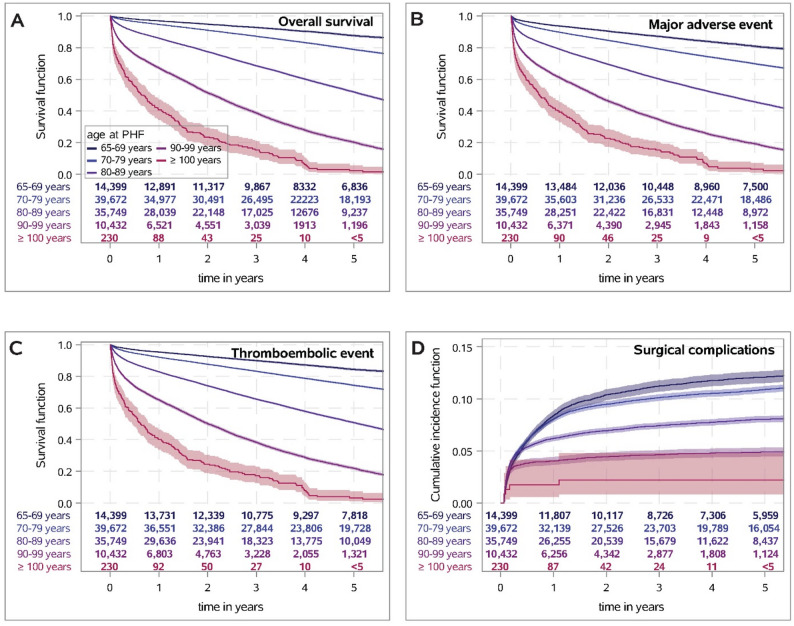
Survival probability with 95% confidence interval (95%CI) determined by Kaplan-Meier estimate for overall survival (**A**), major adverse events (**B**) and thromboembolic events or death (**C**) after proximal humeral fracture depending on age. **D**: Cumulative incidence function with 95%CI determined by Aalen-Johansen estimate for surgical complications during follow-up (starting 21 days after fracture) depending on age, with death being considered as competing risk event

**Fig. 3:**
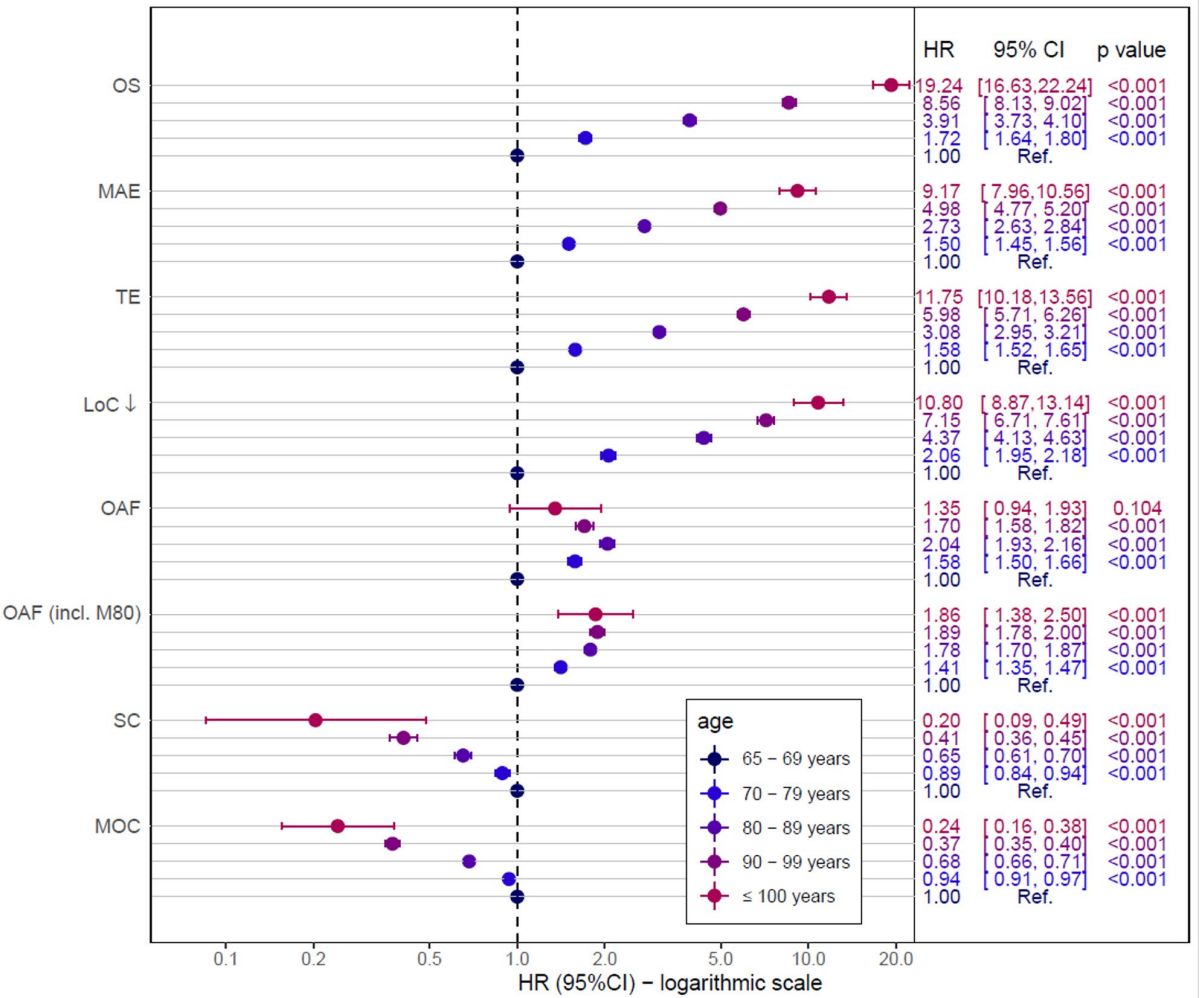
Multivariable regression analysis of all endpoints. Overall survival (OS), major adverse events (MAEs), thromboembolic events (TE) or death and worsening of the level of care (LoC) or death were modeled using Cox proportional hazard models. For secondary osteoporosis-associated fractures (OAFs), surgical or injured-related complications (SC) and minor outpatient complications (MOC), death was considered as a competing risk event and sub-distributional hazard ratios (HRs) were determined by multivariable Fine and Gray models. ^*^ Only patients treated from 2017 onwards were included (*N* = 53,737). Full regression results are presented in Suppl. Table S6

As shown in Fig. 2, the outcome after proximal humeral fracture got worse with increasing age. Five years after proximal humeral fracture, 97.8% (93.1–99.3%) of the centenarians were deceased, 28.7% died 60 days after sustaining a proximal humeral fracture, 44.2% after 180 days and 59.2% after one year. Thus, after adjustment for patients’ risk profile, an age of 100 years and older were associated with a 19-fold higher risk for death compared to patients with an age of 65–69 years (HR: 19.2; 95%CI 16.6–22.2; *p* < 0.001). Similar effects were found for major adverse events, thromboembolic events and worsening of level of care (see Figs. 2 and 3).

By contrast, an age of 100 years and more was associated with lower rates of surgical complications and minor outpatient complication (see Figs. 2 and 3 and suppl. table S2).

### Differences between surgical and non-operative treatment

**Fig. 4:**
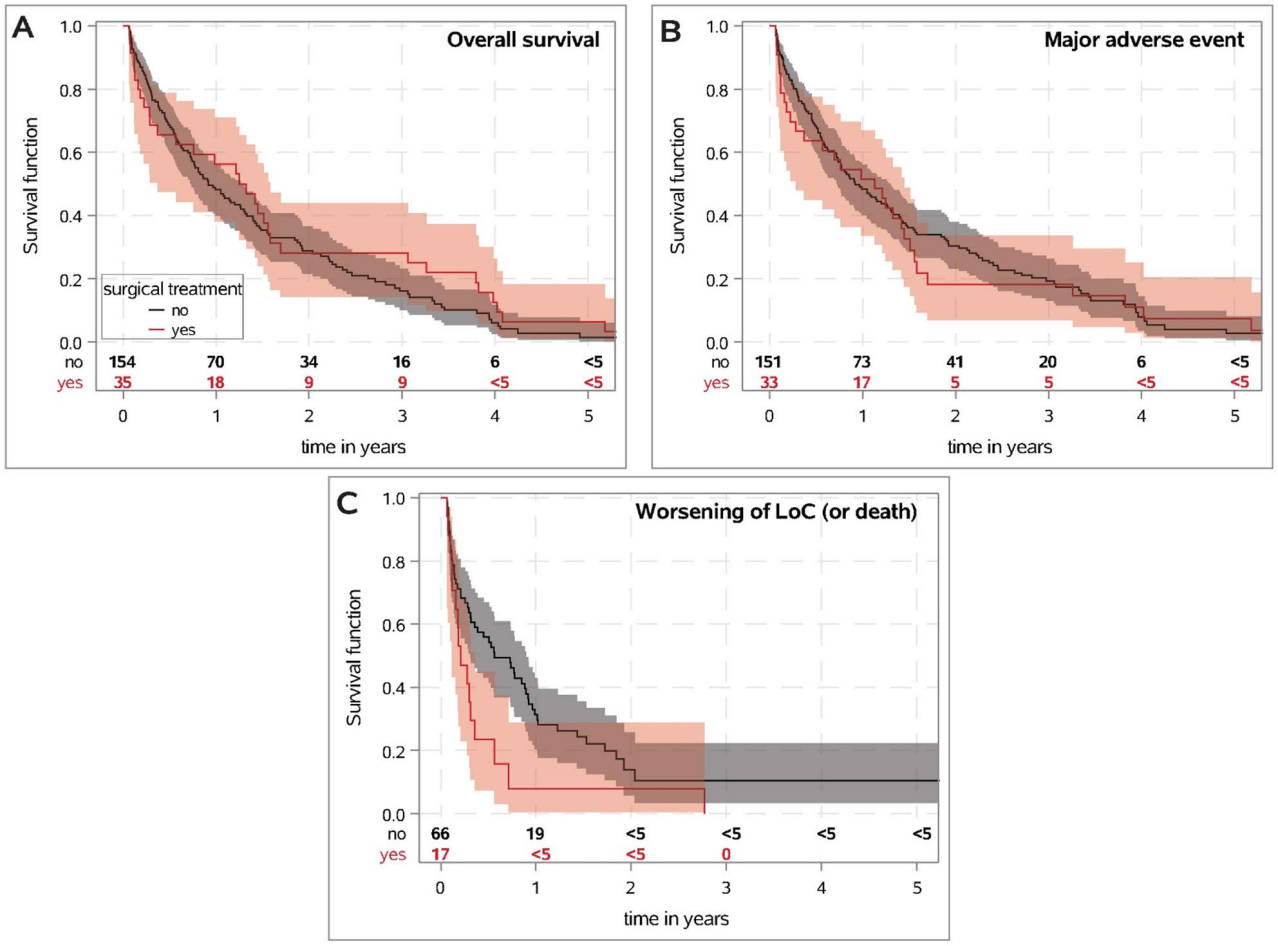
Survival probability with 95% confidence interval determined by Kaplan-Meier estimate for overall survival (**A**), major adverse events (**B**) and worsening of the level of care or death (**C**) comparing surgical and non-operative treatment in centenarians with proximal humeral fracture. Only patients who had an observation period of > 21 days were included, as the allocation of treatment was only completed after this time

Survival function for centenarians with and without surgical treatment were presented in Fig. 4. No differences for the risk of a worse outcome after proximal humeral fracture between surgically and non-surgically treated centenarians could be observed, which was also confirmed in multivariable analyses (see suppl. table S3). However, the association between surgical treatment and surgical complications differs between the age groups (p_int_= 0.047; see suppl. table S3). While surgical treatment was associated with a higher risk of surgical complications in younger patients, no differences between surgical and non-operative treatment were found in centenarians (HR 0.88; 95%CI 0.51–5.25; *p* = 0.891). Moreover, with increasing age, surgical treatment within first 21 days after proximal humeral fracture was decreasingly associated with lower risk for minor outpatient complication (p_int_<0.001; see suppl. Table S3).

### Course of osteoporosis

At the time of proximal humeral fracture, osteoporosis was more often diagnosed in centenarians (44.8% vs. 34.9%), but a pharmaceutical treatment was less often used in those patients within two years before proximal humeral fracture (centenarians 8.7% vs. 12.2%). The higher rates of osteoporosis associated fractures up to five years before proximal humeral fractures seemed unsurprising in this context (see Table 1). In detail, 43% of the centenarians had at least one osteoporosis associated fracture before proximal humeral fracture, compared to 22.7% prior osteoporosis associated fractures in younger patients.

After proximal humeral fracture, differences for the risk of secondary osteoporosis associated fractures could not be observed for centenarians (*p* = 0.104), but for the other age groups (see Fig. 3). The inclusion of ICD code M80 for “osteoporosis with pathological fracture” resulted in a different picture: with increasing age, the risk for secondary osteoporosis associated fractures incl. M80 was increased (≥ 100 years compared to 65–69 years: risk adjusted HR 1.85; 95%CI 1.38–2.49, *p* < 0.001).

## Discussion

This study at hand is – to our knowledge – the first one analyzing proximal humeral fractures in patients aged 100 years and older. The most important findings of this study are that although centenarians are more frequently treated non-operatively, no difference in outcome between operative vs. non-operative treatment could be found. Not surprisingly, the mortality rate after a proximal humeral fracture is elevated for centenarians in comparison to other older patients, although probably not directly associated with proximal humeral fracture.

The majority of the centenarians with a proximal humeral fracture is female. The predominance of female patients can also be seen for the other osteoporotic fractures [5, 15–17]. Furthermore, Hazra et al. showed that the female proportion of centenarians rose significantly from 1990 to 1994 to 2010–2013 [2].This underlines the need for further research on osteoporosis treatment strategies and gender data gap [11].

In this study, more centenarians were treated non-operatively in comparison to patients with an age of 65–99 years. However, every fifth centenarian was treated operatively. A potential selection bias cannot be fully excluded; as frail patients may be less likely to receive surgical treatment or to be admitted to hospital compared to fitter individuals. Such differences in treatment decisions are influenced by multiple clinical and individual factors that cannot be completely captured by administrative data or ICD codes.

However, frailty represents an important factor influencing outcomes in very old patients, and further research should explore its role in this population.

As this is the first study analyzing the treatment of proximal humeral fractures in a centenarian population, it is not possible to compare it to other papers. Hence, the choice of treatment for proximal humeral fractures remains a controversial topic, as in several other studies no outcome difference comparing the non-operative with operative treatment could be observed [18, 19]. Even more for centenarians, the choice of treatment will remain an individual decision for each patient, taking into account patient’s wishes and patient-individual factors.

Regarding the mortality rate as another outcome parameter, the mortality after a proximal humeral fracture rises with patients age. As it is not surprising, that after five years 98% of the centenarians with a proximal humeral fracture are dead, it can be observed that more than half of the patients die within the first year after a proximal humeral fracture. Sumrein et al. also observed an increase of mortality for patients with a proximal humeral fracture with increasing age regarding the population < 100 years. They reported a mortality rate of 20% for patients > 80 years after 12 months and 51% after 48 months, which is still considerably lower than the mortality for centenarians in the present study [20].

Within our data we have observed a one-year mortality of 60% within the centenarians. This is consistent with life expectancy date of the German Federal Statistical Office. For example, in 2022 life expectancy of 99-year-old women was 2.1 years [21].

Nevertheless, the mortality rate for centenarians with a proximal humeral fracture is in line with the survival reported in other studies for osteoporotic hip fractures patients aged > 99 years. In a recent systematic review, Abelleyra Lastoria et al. reported a 1-year mortality of 53.8% after a hip fracture in the centenarian population [4]. Bareclo et al. described a median survival of 1.32 years for patients > 95 years after a hip fracture [22]. Regarding future challenges in an aging society with limited financial resources the reported data will be crucial for cost-effectiveness modelling. However, it will be an ethical issue to enable older patients to be mobilized early with little pain through surgery. In the light of economic challenges this will be a matter of societal discourse.

It is well known that a proximal humeral fracture is one of the most frequent osteoporosis associated fracture [5, 23, 24]. This is also reflected in the results of this study. More than one third of the patients ≥ 65 and almost half of the patients ≥ 100 years of age had a diagnosis of osteoporosis. But the number of patients with an anti-osteoporotic treatment was considerably lower comparing the centenarians to patients aged 65–99 years. This finding was also observed in a Spanish study analyzing the anti-osteoporotic medication in centenarians with hip fracture [3].

In the study of Barceló et al., only a few centenarians sustained a second osteoporotic fracture after their hip fracture [22]. Consistent to the present data, the risk for another osteoporotic associated fracture was not significant for centenarians in contrast to patients aged 65–99 years. However, including the code for “osteoporosis with pathological fracture” the risk increased also for patients ≥ 100 years. Considering the high mortality for the centenarians, it is possible that the patients die before they sustain another fracture. Furthermore, as this study only analyzed insurance data it is unclear, if the code used for “osteoporosis with pathological fracture” reflects all fractures, that occurred after the proximal humeral fracture. If the patient sustained a hip fracture before, a new hip fracture even on the contralateral side will not be adequately taken into account. Additionally, this code might be used for long-term follow-up visits after the proximal humeral fracture and not as a code for a new fracture. One of the main goals of an anti-osteoporotic therapy is to avoid a further osteoporotic fracture. Furthermore, it could be shown, that under an anti-osteoporotic therapy the risk for surgical complications could be reduced [11]. We therefore recommend a consequent anti-osteoporotic therapy also in centenarians.

### Strength and limitations

As a limitation, the BARMER’s policyholders are older and more often female than the national average, resulting in probably higher ratio of older patients. Moreover, due to the low number of patients with an age of 100 years and older, the results must be interpreted accordingly. However, the presented study had – to our knowledge – one of the largest cohorts with an age-related fracture and an age of 100 years and more. In general, limiting the presented results, neither clinical data nor the cause of treatment decision were available and the data were initially collected for financial purposes not for scientific research. Further research is needed to check the observed results. The strengths of the study were given by the high number of observed patients and the long follow-up period up to 12 years.

## Conclusions

The majority of centenarians sustaining a proximal humeral fracture is female. The choice of treatment remains challenging and must be based on individual factors. The most common treatment was the non-operative therapy. Nevertheless, also the operative therapy is associated with a low complication rate. However, as expected the mortality rate is high with almost 60% of deceased patients after one year. 

## Supplementary Information

Supplementary Material 1.

## Data Availability

The authors confirm that the data utilized in this study cannot be made available in the manuscript, the supplemental files, or in a public repository due to German data protection laws (‘Bundesdatenschutzgesetz’, BDSG). They are stored on a server of the BARMER Institute for Health System Research, to facilitate replication of the results. In general, access to data of statutory health insurance funds for research purposes is possible only under the conditions defined in German Social Law (SGB V § 287).

## Abbreviations

PHF: Proximal humeral fracture
MAE: Major adverse event
RTSA: Reverse total shoulder arthroplasty
DRG: German Diagnosis Related Groups
OS: Overall survival
TE: Thromboembolic event
SC: Surgical–related complication
OAF: Osteoporosis–associated fracture
LoC: Level of care
MOC: Minor outpatient complication
BDSG: Bundesdatenschutzgesetz
HR: Hazard ratio
CI: Confidence interval
