# Supplementary material for: Centenarians with proximal humeral fracture
Source: BMC Geriatr. 2025 Dec 2;26:19. doi: 10.1186/s12877-025-06820-w (PMC12777488; doi:10.1186/s12877-025-06820-w)
Supplement: Supplementary file 1 — Supplementary Material 1. [file 12877_2025_6820_MOESM1_ESM.docx]

**Supplements**

**Appendix Figure S1: Consort flow chart.**


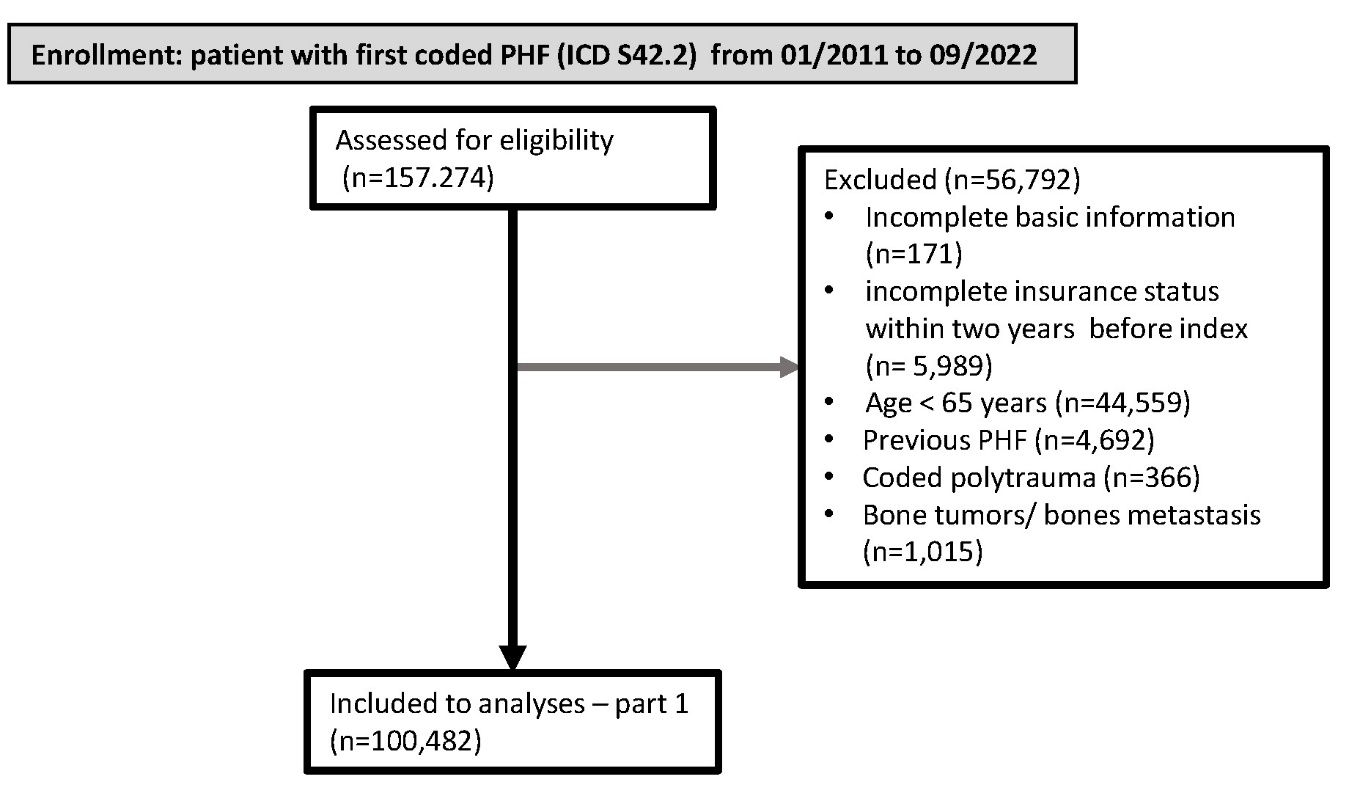


**Appendix Table S1:** Definition of all variables. LPF – locked pate fixation, PHF – proximal humeral fracture, RTSA – reverse total shoulder arthroplasty.

^1^Data collection based on outpatient and inpatient information in the period of two years before the index event (i.e. first coded diagnosis of PHF). Exception: Rotator cuff rupture – only if coded at the index event.

^2^Only based on outpatient data after PHF.

| Variable | Classification | Codes |
| --- | --- | --- |
| Proximal humeral fracture | ICD | **S42.2** |
| Simple fracture LPF | OPS | **5-793.k1, 5-793.31** |
| Multi-fragmented LPF | OPS | **5-794.21, 5-794.k1** |
| RTSA | OPS | **5-824.21** |
| Other fracture fixation | OPS | **5-790.01**, 5-790.11, 5-790.21, 5-790.31, 5-790.41, 5-790.51, 5-790.61, 5-790.71, 5-790.81,  5-790.91, 5-790.d1, 5-790.m1, 5-790.n1, 5-790.p1, 5-790.x1, **5-793.11**, 5-793.21, 5-793.41,  5-793.51, 5-793.61, 5-793.71, 5-793.81, 5-793.91, 5-793.a1, 5-793.b1, 5-793.c1, 5-793.g1,  5-793.m1, 5-793.n1, 5-793.x1, **5-794.01**, 5-794.11,5-794.31, 5-794.41, 5-794.51, 5-794.61,  5-794.71, 5-794.81, 5-794.a1, 5-794.b1, 5-794.c1, 5-794.g1, 5-794.m1, 5-794.n1, 5-794.x1,  **5-824.00**, 5-824.01, 5-824.0x, 5-824.20 |
| Comorbidities at index^1^ | | |
| Alcohol abuses | ICD | **E**24.4, **F**10, **G**31.2, G62.1, G72.1, **I**42.6, **K**29.2, K70, K85.2, K86.0, **T**51.0, T51.9 |
| Atherosclerosis | ICD | I70 |
| Atrial fibrillation and flutter | ICD | I48 |
| Bone tumor/metastasis | ICD | C40.0, C79.5, C79.86, C79.9 |
| Cancer | ICD | C |
| Chronic kidney disease | ICD | N18, N19 |
| Chronic polyarthritis | ICD | M05, M06 |
| Congestive heart failure | ICD | I50 |
| Coronary heart disease | ICD | I25 |
| Dementia | ICD | **F**00, F01, F02, F051, **G**30, G31.1 |
| Diabetes mellitus | ICD | E10 – E14 |
| Hypertension | ICD | I10 – I15 |
| Infection | ICD | **M**86.01, M86.11, M86.21, M86.31, M86.41, M86.51, M86.61, M86.81, M86.91, **T**84.5, T84.6, T84.7 |
| Nicotine abuses | ICD | F17 |
| Obesity | ICD | E66 |
| Osteoporosis | ICD | M80 – M85 |
| Parkinson | ICD | G20 |
| Polytrauma | ICD, OPS, DRG | ICD: T07; OPS: 5-982, or coded DRG started with “W” |
| Rotator cuff rupture | ICD | M75.1, S46.0 |
| Omarthrosis | ICD | M25.51 |
| Previous stroke and other cerebrovascular disease | ICD | I60 – I69 |
| Pharmaceutical therapy | | |
| Any anticoagulant | ATC | B01AA, B01AB, B01AC, B01AE, B01AF, B01AX |
| Vitamin D or calcium | ATC | A11CC |
| Bisphosphonates | ATC | M05BA, M05BB |
| Any osteoporosis pharmacotherapy | ATC | Vitamin D/Calcium or Bisphosphonates |
| Osteoporosis associated fractures | | |
| Distal radius | ICD | S52.2, S52.6 |
| Proximal femur | ICD | S72.0, S72.1 |
| vertebral fracture | ICD | S12.0, S12.1, S12.2, S12.7, S12.9, S22.0, S32.0 |
| pelvic ring fractures | ICD | S32.1, S32.2 S32.3, S32.5, S32.8.1 |
| Osteoporosis with pathologic fracture | ICD | M80 |
| General complications^2^ | | |
| Acute liver failure | ICD | K72.0, K72.7, K72.9 |
| Acute myocardial infarction | ICD | I21, I22 |
| Acute renal failure | ICD | N17 |
| Acute respiratory distress syndrome | ICD | J80 |
| Cardiac arrest | ICD | I46 |
| Deep vein thrombosis | ICD | I80.1, I80.2, I82.2, I82.3 |
| Pulmonary embolism | ICD | I26 |
| Sepsis | ICD | A41, A40, B37.7, R65.0, R65.1, R65.9, R57.2 |
| Stroke | ICD | I60 – I64 |
| Injured-related or surgical complications^2^ | | |
| Failure of non-surgical treatment, any | OPS | **5-78**5.01, 5-785.11, 5-785.21, 5-785.51, 5-785.61, 5-785.71, **5-789**.31, **5-78a**.01, 5-78a.11,  5-78a.21, 5-78a.31, 5-78a.41, 5-78a.51, 5-78a.61, 5-78a.71, 5-78a.90, 5-78a.c1, 5-78a.g1,  5-78a.k1, **5-790.01,** 5-790.11, 5-790.21, 5-790.31, 5-790.41, 5-790.51, 5-790.61, 5-790.71,  5-790.81, 5-790.91, 5-790.d1, 5-790.m1, 5-790.n1, 5-790.p1, 5-790.x1, **5-793**.01, 5-793.11,  5-793.21, 5-793.31, 5-793.41, 5-793.51, 5-793.81, 5-793.91, 5-793.c1, 5-793.g1, 5-793.a1,  5-793.b1, 5-793.h1, 5-793.k1, 5-793.m1, 5-793.n1, 5-793.x1, **5-794**.01, 5-794.11, 5-794.21,  5-794.31, 5-794.41, 5-794.51, 5-794.61, 5-794.71, 5-794.81, 5-794.a1, 5-794.b1, 5-794.c1,  5-794.g1, 5-794.k1, 5-794.m1, 5-794.n1, 5-794.x1, **5-824**.00**,** 5-824.01, 5-824.21, 5-824.0x |
| Upper limb amputation, ipsilateral (shoulder or upper arm) | OPS | **5-862**.1, 5-862.2 |
| Delayed union (LPF and non-surgical treatment; if coded within six months) | OPS | **5-781**.a1, **5-782**.11, 5-782.21, 5-782.31, 5-782.41, 5-782.51, 5-782.61, 5-782.a1, 5-782.b1,  **5-784**.01, 5-784.11, 5-784.21, 5-784.31, 5-784.41, 5-784.71, 5-784.81, 5-784.b1 |
| Infection | OPS | **5-780**.41, 5-780.51, 5-780.61, 5-780.71, 5-780.81, 5-780.91, **5-800**.20, 5-800.30, 5-800.a0,  5-800.b0, **5-810**.10, 5-810.70, 5-810.80, **8-989**, 8-989.0, 8-989.1, 8-989.2, 8-989.3, 8-989.4,  8-989.5, 8-989.6 |
| Joint damage / cartilage damage (LPF and non-surgical treatment) | OPS | 5-780.31, 5-784.51, 5-784.61, 5-800.80, 5-810.40, 5-812.00, 5-812.30, 5-812.90, 5-812.a0,  5-812.e0, 5-812.f0, 5-812.g0, 5-812.h0, 5-812.k0, 5-812.m0 |
| Luxation | OPS | **8-201.0** |
| Malunion (LPF and non-surgical treatment) | OPS | **5-781**.01, 5-781.11, 5-781.21, 5-781.31, 5-781.41, 5-781.51, 5-781.61, 5-781.81, 5-781.91 |
| Nerve injury | OPS | **5-040**.1, 5-040.2, 5-040.3, **5-041**.1, 5-041.2, 5-041.3, **5-044**.1, 5-044.2, 5-044.3, **5-045**.1,  5-045.2, 5-045.3, **5-046**.1, 5-046.2, 5-046.3, **5-047**.1, 5-047.2, 5-047.3, **5-048**.1, 5-048.2,  5-048.3, **5-049**.1, 5-049.2, 5-049.3, **5-04b**.1, 5-04b.2, 5-04b.3, **5-050**.1, 5-050.2, 5-050.3,  **5-051**.1, 5-051.2, 5-051.3, **5-052**.1, 5-052.2, 5-052.3, **5-053**.1, 5-053.2, 5-053.3, **5-054**.1,  5-054.2, 5-054.3, **5-055**.1, 5-055.2, 5-055.3, **5-056**.1, 5-056.2, 5-056.3, **5-057**.1, 5-057.2,  5-057.3 |
| Non-union / Pseudoarthrosis (LPF and non-surgical treatment; if coded after six months) | OPS | **5-781**.a1, **5-782**.11, 5-782.21, 5-782.31, 5-782.41, 5-782.51, 5-782.61, 5-782.a1, 5-782.b1,  **5-784**.01, 5-784.11, 5-784.21, 5-784.31, 5-784.41, 5-784.71, 5-784.81, 5-784.b1 |
| Osteonecrosis (LPF and non-surgical treatment) | ICD | **M87.21, M87.22, M87.32, M87.82, M87.92** |
| Postoperative stiffness, Adhesive capsulitis, Frozen shoulder | OPS | **5-800**.60, 5-800.c0, **5-810**.20, 5-810.90 |
| Vascular injury | OPS | **5-388**.11, 5-388.12, **5-395**.11, 5-395.12, **5-397**.11, 5-397.12 |
| Secondary surgery, open (LPF, non-surgical treatment) | OPS | **5-780**.01, 5-780.11, 5-780.21, 5-780.31, 5-780.61, 5-780.x1, **5-782**.11, 5-782.21, 5-782.31,  5-782.41, 5-782.51, 5-782.62, 5-782.72, 5-782.82, 5-782.92, 5-782.a1, **5-784**.01, 5-784.11,  5-784.21, 5-784.31, 5-784.41, 5-784.51, 5-784.61, 5-784.71, 5-784.81, 5-784.a1, 5-784.b1,  **5-785**.01, 5-785.11, 5-785.21, 5-785.31, 5-785.41, 5-785.51, 5-785.61, 5-785.71, **5-789**.b1,  5-789.c1, **5-794**.01, 5-794.11, 5-794.21, 5-794.31, 5-794.41, 5-794.71, 5-794.81, 5-794.k1,  **5-800**.10, 5-800.30, 5-800.40, 5-800.50, 5-800.70, 5-800.80, 5-800.90, 5-800.x0, **5-801**.00,  5-801.30, 5-801.40, 5-801.b0, 5-801.c0, 5-801.g0, 5-801.h0, 5-801.k0, 5-801.m0, 5-801.n0,  5-801.p0, **5-805**.0, 5-805.1, 5-805.2, 5-805.3, 5-805.4, 5-805.5, 5-805.6, 5-805.7, 5-805.8,  5-805.9, 5-805.a, **5-850**.01,5-850.11, 5-850.21, 5-850.31, 5-850.41, 5-850.51, 5-850.61,  5-850.71, 5-850.81, 5-850.91, 5-850.a1, 5-850.b1, 5-850.c1, 5-850.d1, 5-850.e1, 5-850.f1,  5-850.g1, 5-850.h1, 5-850.j1, 5-850.x1, **5-851**.11, 5-851.21, **5-852**.01, 5-852.11, **5-853**.01,  5-853.11, **5-855**.01, 5-855.11, 5-855.21, 5-855.51, 5-855.61, 5-855.71, 5-855.81, 5-855.91,  5-855.a1, **5-859**.01, 5-859.11, **5-862**.1, 5-862.2, **5-892**.06, 5-892.07, 5-892.16, 5-892.17,  **5-896**.06, 5-896.16, 5-896.26 |
| Secondary surgery, open (RTSA) | OPS | **5-780**.01, 5-780.11, 5-780.21, 5-780.31, 5-780.61, 5-780.x1, **5-782**.11, 5-782.21, 5-782.31,  5-782.41, 5-782.51, 5-782.62, 5-782.72, 5-782.82, 5-782.92, 5-782.a1, **5-785**.01, 5-785.11,  5-785.21, 5-785.31, 5-785.41, 5-785.51, 5-785.61, 5-785.71, **5-789**.b1, **5-791**.02, 5-791.12,  5-791.22, **5-792**.02, 5-792.12, 5-792.22, 5-792.k2, **5-800**.10, 5-800.30, 5-800.40, 5-800.50,  5-800.70, 5-800.80, 5-800.90, 5-800.x0, **5-850**.01, 5-850.11, 5-850.21, 5-850.31, 5-850.41,  5-850.51, 5-850.61, 5-850.71, 5-850.81, 5-850.91, 5-850.a1, 5-850.b1, 5-850.c1, 5-850.d1,  5-850.e1, 5-850.f1, 5-850.g1, 5-850.h1, 5-850.j1, 5-850.x1, **5-859**.01, 5-859.11, **5-862**.1,  5-862.2, **5-892**.06, 5-892.07, 5-892.16, 5-892.17, **5-896**.06, 5-896.16, 5-896.26 |
| Secondary arthroscopy (LPF, non-surgical treatment) | OPS | **5-782**.b1, **5-784**.c1, 5-784.d1, 5-784.e1, 5-784.f1, **5-810**.00, 5-810.10, 5-810.20, 5-810.40,  5-810.50, 5-810.70, 5-810.80, 5-810.90, **5-811**.20, 5-811.30, 5-811.40, **5-812**.00, 5-812.30,  5-812.40, 5-812.90, 5-812.a0, 5-812.e0, 5-812.f0, 5-812.g0, 5-812.h0, 5-812.k0, 5-812.m0,  **5-814**.0, 5-814.1, 5-814.2, 5-814.3, 5-814.4, 5-814.5, 5-814.6, 5-814.7, 5-814.8, 5-814.9,  5-814.b, 5-814.c, 5-814.d, 5-814.e, **5-819**.00, 5-819.10, 5-819.20 |
| Secondary arthroscopy (RTSA) | OPS | **5-782**.b1, **5-810**.00, 5-810.10, 5-810.20, 5-810.40, 5-810.50, 5-810.70, 5-810.80, 5-810.90,  **5-811**.20, 5-811.30, 5-811.40, **5-814**.b, 5-814.c, 5-814.d, 5-814.e, **5-819**.00, 5-819.10,  5-819.20 |
| Revision (LPF) | OPS | **5-78**5.01, 5-785.11, 5-785.21, 5-785.51, 5-785.61, 5-785.71 **5-789**.31, 5-78a.01, 5-78a.11,  5-78a.21, 5-78a.31, 5-78a.41, 5-78a.51, 5-78a.61, 5-78a.71, 5-78a.90, 5-78a.c1, 5-78a.g1,  5-78a.k1, **5-793**.01, 5-793.11, 5-793.21, 5-793.31, 5-793.41, 5-793.51, 5-793.81, 5-793.91,  5-793.c1, 5-793.g1, 5-793.a1, 5-793.b1, 5-793.h1, 5-793.k1, 5-794.01, **5-794**.11, 5-794.21,  5-794.31, 5-794.41, 5-794.71, 5-794.81, 5-794.k1, **5-824**.01, 5-824.21 |
| Revision (RTSA) | OPS | **5-785**.01, 5-785.11, **5-787**.01, 5-787.11, 5-787.21, 5-787.31, 5-787.k1, **5-789**.31, **5-78a**.01,  5-78a.11, 5-78a.21, 5-78a.k1, **5-810**.40, **5-824**.21, **5-825**.00, 5-825.1 (only 2010-2012),  5-825.21, 5-825.8, 5-825.k, 5-825.k0, 5-825.k1, 5-825.kx |
| Resection arthroplasty (for spacer placement) | OPS | **5-829.3** |
| Secondary arthroplasty (LPF and non-surgical treatment) | OPS | **5-824**.0, 5-824.20, 5-824.21 |
| Arthrolysis (LPF and non-surgical treatment) | OPS | **5-800**.60, **5-810**.20, 5-810.90 |
| Decompression of subacromial space (LPF and non-surgical treatment) | OPS | **5-814.3** |
| Debridement (LPF and non-surgical treatment) | OPS | **5-819**.10 |
| Endpoints | | |
| Major adverse events |  | resuscitation, cardiac arrest, myocardial infarction, stroke, acute renal failure, acute liver failure, acute respiratory distress syndrome, sepsis or death from any cause |
| Thromboembolic event |  | Deep vein thrombosis, pulmonary embolism, ischemic stroke or death from any cause |
| Surgical complications |  | Adhesive capsulitis, arthrolysis, conversion debridement, decompression of subacromial space, frozen shoulder, infection, infection with antibiotic-resistant germs, joint damage/cartilage damage, luxation, delayed union, non-union/ pseudoarthrosis, malunion, nerve injury, vascular injury, osteonecrosis, postoperative stiffness, secondary arthroplasty, secondary arthroscopy, secondary surgery (open) including revision surgery, upper limb amputation |
| Minor outpatient complications  (LPF) | ICD | **G**56.1, G56.2, G56.3, **I**80.80, I80.81, **M**00.01, M00.11, M00.21, M00.81, M00.91, M13.11, M13.81, M13.91, M19.11, M24.01, M24.11, M24.21, M24.41, M24.31, M24.51, M24.61, M25.11, M25.21, M25.31, M25.41, M25.51, M25.61, M25.71, M61.01, M62.21, M62.22, M62.41, M62.51, M62.61, M65.81, M65.91, M75.0, M75.1, M75.2, M75.4, M75.5, M84.21, M84.31, M86.01, M86.11, M86.21, M86.31, M86.41, M86.51, M86.61, M86.81, M86.91, M87.21, M87.22M87.31, M87.81, M87.91, M89.51, M96.6, **T**79.60, T84.10, T84.5, T84.6, T84.7 |
| Minor outpatient complications  (RTSA) | ICD | **G**56.1, G56.2, G56.3, **I**80.80, I80.81, **M**00.01, M00.11, M00.21, M00.81, M00.91, M24.21, M24.41, M24.31, M24.51, M24.61, M25.11, M25.21, M25.31, M25.41, M25.51, M25.61, M25.71, M61.01, M62.21, M62.41, M62.51, M62.61, M65.81, M65.91, M75.0, M75.1, M75.2, M75.4, M75.5, M84.31, M86.01, M86.11, M86.21, M86.31, M86.41, M86.51, M86.61, M86.81, M86.91, M96.6, **T**79.60, T81.4, T84.5, T84.6, T84.7, T84.00 |
| Minor outpatient complications  (non-surgical treatment) | ICD | **G**56.1, G56.2, G56.3, **I**80.80, I80.81, **M**00.01, M00.11, M00.21, M00.81, M00.91, M13.11, M13.81, M13.91, M19.11, M24.01, M24.11, M24.21, M24.41, M24.31, M24.51, M24.61, M25.11, M25.21, M25.31, M25.41, M25.51, M25.61, M25.71, M61.01, M62.21, M62.22, M62.41, M62.51, M62.61, M65.81, M65.91, M75.0, M75.1, M75.2, M75.4, M75.5, M84.21, M84.31, M86.01, M86.11, M86.21, M86.31, M86.41, M86.51, M86.61, M86.81, M86.91, M87.21, M87.22, M87.32, M87.82, M87.92, M89.51, M96.6, **T**79.60 |

**Appendix Figure S2**: Ratio of patients with surgical treatment and outpatient diagnosis depending on age.


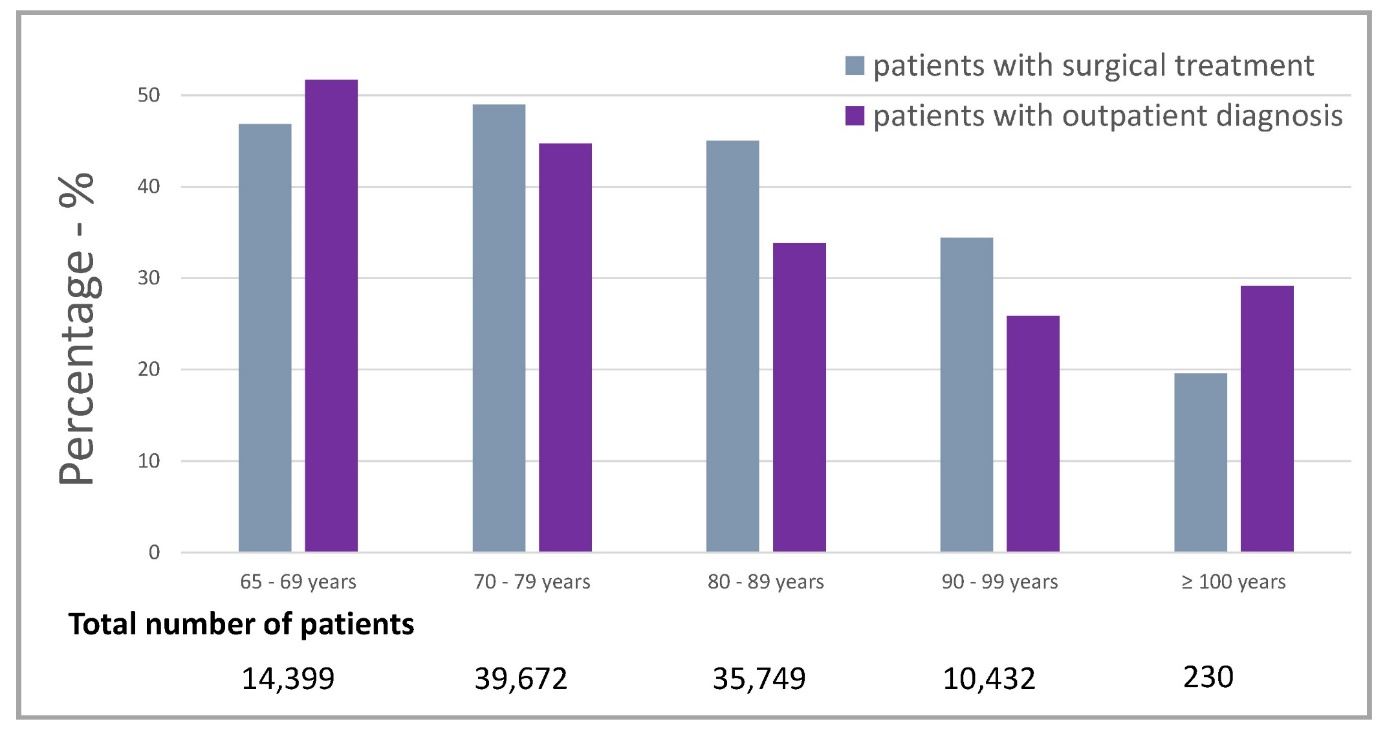


**Appendix Table S2:** Event rates with 95% confidence interval (95%CI) for primary and secondary outcomes. For overall mortality (OM); major adverse events (MAEs) and thrombo-embolic events (TEs), event rates were given as 1- survival function S(t) determined by Kaplan-Meier estimate. For surgical complications (SC), secondary osteoporosis associated fractures (OAF) and minor outpatient complications, death was considered as a competing risk event and event rates were given by cumulative incidence function, determined by Aalen-Johansen estimate.

| Endpoint | Entire cohort | Age 65 – 69 years | 70-79 years | 80-89 years | 90-99 years | ≥ 100 years |
| --- | --- | --- | --- | --- | --- | --- |
| OM:  1 year rate  2 years rate  3 years rate  4 years rate  5 years rate  total no. of events | 11.1% (10.9 – 11.3%)  17.5% (17.3 – 17.8%)  23.8% (23.5 – 24.1%)  29.8% (29.5 – 30.1%)  35.5% (35.1 – 35.8%)  38,299 | 3.1% (2.8 – 3.4%)  5.2% (4.8 – 5.6%)  7.3% (6.8 – 7.7%)  9.7% (9.2 – 10.3%)  12.4% (11.7 – 13.0%)  2,093 | 5.4% (5.1 – 5.6%)  9.0% (8.7 – 9.3%)  12.8% (12.5 – 13.2%)  16.9% (16.5 – 17.3%)  21.2% (20.7 – 21.6%)  10,439 | 14.0% (13.7 – 14.4%)  22.6% (22.2 – 23.1%)  31.5% (31.0 – 32.0%)  40.0% (39.4 – 40.5%)  48.1% (47.5 – 48.7%)  17,568 | 32.8% (31.9 – 33.7%)  48.6% (47.6 – 49.5%)  61.3% (60.3 – 62.3%)  72.2% (71.2 – 73.2%)  79.8% (78.9 – 80.7%)  7,993 | 59.2% (52.5 – 65.4%)  76.5% (70.1 – 81.7%)  84.6% (78.6 – 89.0%)  93.8% (88.9 – 96.6%)  97.8% (93.1 – 99.3%)  206 |
| MAE:  1 year rate  2 years rate  3 years rate  4 years rate  5 years rate  total no. of events | 16.6% (16.4 – 16.9%)  24.1% (23.8 – 24.4%)  30.9% (30.6 – 31.2%)  36.9% (36.6 – 37.2%)  42.2% (41.9 – 42.5%)  47,234 | 6.4% (6.0 – 6.8%)  9.6% (9.2 – 10.1%)  12.8% (12.2 – 13.3%)  16.0% (15.3 – 16.6%)  19.1% (18.4 – 19.8%)  3,418 | 10.3% (10.0 – 10.6%)  15.4% (15.1 – 15.8%)  20.6% (20.2 – 21.0%)  25.6% (25.1 – 26.0%)  30.2% (29.7 – 30.7%)  14,708 | 21.0% (20.6 – 21.4%)  30.6% (30.1 – 31.1%)  39.5% (39.0 – 40.0%)  47.3% (46.7 – 47.8%)  54.3% (53.7 – 54.9%)  20,513 | 39.0% (38.0 – 39.9%)  53.8% (52.8 – 54.8%)  65.0% (64.1 – 66.0%)  74.1% (73.2 – 75.0%)  80.7% (79.9 – 81.6%)  8,386 | 60.9% (54.2 – 66.9%)  77.4% (71.2 – 82.3%)  84.7% (79.0 – 89.0%)  93.1% (87.6 – 96.2%)  96.9% (91.8 – 98.9%)  209 |
| TE:  1 year rate  2 years rate  3 years rate  4 years rate  5 years rate  total no. of events | 13.6% (13.4 – 13.8%)  20.5% (20.3 – 20.8%)  26.8% (26.5 – 27.1%)  32.6% (32.3 – 32.9%)  37.9% (37.5 – 38.2%)  43,140 | 4.7% (4.3 – 5.0%)  7.5% (7.0 – 7.9%)  10.1% (9.6 – 10.6%)  12.8% (12.3 – 13.4%)  15.6% (14.9 – 16.2%)  2,841 | 7.9% (7.6 – 8.2%)  12.4% (12.0 – 12.7%)  16.8% (16.4 – 17.2%)  21.3% (20.9 – 21.7%)  25.7% (25.2 – 26.1%)  12,892 | 17.1% (16.7 – 17.5%)  26.0% (25.5 – 26.4%)  34.4% (33.9 – 34.9%)  42.1% (41.6 – 42.7%)  49.3% (48.7 – 49.9%)  19,047 | 34.8% (33.9 – 35.7%)  49.9% (48.9 – 50.9%)  61.7% (60.7 – 62.6%)  71.3% (70.3 – 72.2%)  78.1% (77.2 – 79.0%)  8,154 | 60.0% (53.3 – 66.0%)  75.6% (69.3 – 80.7%)  82.6% (76.7 – 87.2%)  92.1% (86.4 – 95.5%)  96.8% (91.5 – 98.8%)  206 |
| SC starting 21days after PHF  1 year rate  2 years rate  3 years rate  4 years rate  5 years rate  total no. of events | 7.0% (6.9 – 7.2%)  8.2% (8.0 – 8.3%)  8.8% (8.6 – 8.9%)  9.1% (8.9 – 9.3%)  9.4% (9.2 – 9.6%)  9,316 | 8.4% (7.9 – 8.9%)  10.4% (9.9 – 10.9%)  11.2% (10.7 – 11.8%)  11.8% (11.2 – 12.3%)  12.1% (11.5 – 12.7%)  1,711 | 8.1% (7.8 – 8.3%)  9.5% (9.2 – 9.8%)  10.1% (9.8 – 10.4%)  10.5% (10.2 – 10.8%)  10.9% (10.6 – 11.2%)  4,335 | 6.2% (6.0 – 6.5%)  6.9% (6.7 – 7.2%)  7.4% (7.2 – 7.7%)  7.6% (7.5 – 8.0%)  8.0% (7.6 – 8.3%)  2,770 | 4.0% (3.7 – 4.4%)  4.4% (4.1 – 4.9%)  4.7% (4.3 – 5.1%)  4.8% (4.4 – 5.2%)  4.9% (4.5 – 5.3%)  495 | 1.7% (0.6 – 4.1%)  2.2% (0.8 – 4.8%)  2.2% (0.8 – 4.8%)  2.2% (0.8 – 4.8%)  2.2% (0.8 – 4.8%)  5 |
| Minor outpatient  1 year rate  2 years rate  3 years rate  4 years rate  5 years rate  total no. of events | 23.6% (23.3 – 23.8%)  27.0% (26.7 – 27.3%)  29.0% (28.7 – 29.3%)  30.5% (30.2 – 30.8%)  31.8% (31.5 – 32.1%)  31,319 | 29.8% (29.0 – 30.5%)  34.1% (33.3 – 34.9%)  36.3% (35.5 – 37.2%)  38.5% (37.6 – 39.3%)  40.1% (39.2 – 40.9%  5,722 | 27.6% (27.2 – 28.1%)  31.7% (31.2 – 32.1%)  34.0% (33.5 – 34.4%)  35.9% (35.4 – 36.4%)  37.5% (37.0 – 38.0%)  14,813 | 20.2% (19.8 – 20.6%)  23.2% (22.7 – 23.6%)  25.1% (24.6 – 25.5%)  26.3% (25.8 – 26.8%)  27.2% (26.7 – 27.7%)  9,300 | 11.3% (10.7 – 12.0%)  12.8% (12.2 – 13.5%)  13.7% (13.0 – 14.4%)  14.1% (13.5 – 14.8%)  14.5% (13.8 – 15.2%)  1,464 | 7.4% (4.5 – 11.3%)  8.4% (5.2 – 12.5%)  8.4% (5.2 – 12.5%)  8.4% (5.2 – 12.5%)  8.4% (5.2 – 12.5%)  20 |
| At least one secondary OAFs  1 year rate  2 years rate  3 years rate  4 years rate  5 years rate  total no. of events | 5.8% (5.6 – 5.9%)  9.8% (9.6 – 10.0%)  13.2% (13.0 – 13.4%)  16.2% (16.0 – 16.5%)  18.9% (18.7 – 19.2%)  19,317 | 2.6% (2.4 – 2.9%)  4.8% (4.4 – 5.1%)  6.9% (6.4 – 7.3%)  9.0% (8.5 – 9.5%)  11.1% (10.6 – 11.7%)  1,784 | 4.6% (4.4 – 4.9%)  8.1% (7.8 – 8.4%)  11.4% (11.1 – 11.8%)  14.5% (14.1 – 14.9%)  17.5% (17.1 – 17.9%)  7,676 | 7.6% (7.3 – 7.8%)  12.7% (12.3 – 13.0%)  16.9% (16.5 – 17.3%)  20.5% (20.1 – 21.0%)  23.6% (23.1 – 24.1%)  7,876 | 8.4% (7.9 – 9.0%)  13.4% (12.8 – 14.1%)  16.5% (15.7 – 17.2%)  18.5% (17.7 – 19.3%)  20.1% (19.3 – 21.0%)  1,949 | 7.6% (4.6 – 11.5%)  13.0% (8.9 – 18.0%)  14.1% (9.8 – 19.2%)  14.7% (10.3 – 19.9%)  14.7% (10.3 – 19.9%)  32 |
| At least one secondary OAFs (incl. M80)  1 year rate  2 years rate  3 years rate  4 years rate  5 years rate  total no. of events | 14.6% (14.3 – 14.8%)  19.9% (19.7 – 20.2%)  24.4% (24.1 – 24.7%)  28.5% (28.2 – 28.9%)  32.4% (32.0 – 32.7%)  29,133 | 8.0% (7.6 – 8.5%)  11.0% (10.5 – 11.5%)  13.8% (13.2 – 14.4%)  16.5% (15.8 – 17.1%)  19.2% (18.5 – 19.9%)  2,883 | 12.9% (12.6 – 13.2%)  17.7% (17.3 – 18.1%)  21.8% (21.4 – 22.3%)  25.7% (25.2 – 26.2%)  29.5% (29.0 – 30.0%)  11,656 | 17.9% (17.5 – 18.3%)  24.5% (24.1 – 25.0%)  30.1% (29.6 – 30.6%)  35.5% (34.9 – 36.1%)  40.1% (39.4 – 40.8%)  11,606 | 19.2% (18.4 – 20.0%)  27.4% (26.4 – 28.4%)  33.5% (32.4 – 34.7%)  38.9% (37.4 – 40.1%)  44.0% (42.4 – 45.6%)  2,940 | 15.5% (10.8 -21.0%)  31.7% (22.4 – 41.4%)  37.3% (25.8 – 48.8%)  40.8% (27.9 – 53.3%)  40.8% (27.9 – 53.3%)  48 |

**Appendix Table S3:** Multivariable regression analyses to account for differences between surgical and non-surgical treatment depending on age. Modelling was performed using multivariable Cox regression models or Fine & Gray models for competing risk events including age group, sex, time-dependent treatment group with 21 days after proximal humeral fracture (PHF) and an interaction term between treatment and age group. For surgical or injured-related complication (SC) and minor outpatient complications (MOC), death was considered as a competing risk event and sub-distributional hazard ratios (HRs) are presented. CI – confidence interval, LoC – Level of care.

| Age | HR comparing surgical treatment (yes vs no) | 95% CI | P value | Interaction p value: p_int_ |
| --- | --- | --- | --- | --- |
| Overall survival  65 – 69 years  70 – 79 years  80 – 89 years  90 – 99 years  ≥ 100 years | 0.84  0.83  0.84  0.85  0.98 | 0.77 – 0.92  0.80 – 0.86  0.81 – 0.86  0.81 – 0.89  0.70 – 1.37 | <0.001  <0.001  <0.001  <0.001  0.895 | 0.820 |
| Major adverse events  65 – 69 years  70 – 79 years  80 – 89 years  90 – 99 years  ≥ 100 years | 0.71  0.71  0.71  0.76  0.94 | 0.66 – 0.76  0.69 – 0.73  0.69 – 0.74  0.73 – 0.79  0.67 – 1.32 | <0.001  <0.001  <0.001  <0.001  0.711 | 0.061 |
| Thromboembolic events  65 – 69 years  70 – 79 years  80 – 89 years  90 – 99 years  ≥ 100 years | 0.67  0.70  0.70  0.73  0.80 | 0.62 – 0.72  0.67 – 0.72  0.68 – 0.72  0.70 – 0.77  0.57 – 1.12 | <0.001  <0.001  <0.001  <0.001  0.199 | 0.205 |
| SC  65 – 69 years  70 – 79 years  80 – 89 years  90 – 99 years  ≥ 100 years | 1.43  1.24  1.22  1.43  0.89 | 1.30 – 1.58  1.17 – 1.32  1.13 – 1.32  1.20 – 1.71  0.15 – 5.16 | <0.001  <0.001  <0.001  <0.001  0.894 | 0.047 |
| MOC  65 – 69 years  70 – 79 years  80 – 89 years  90 – 99 years  ≥ 100 years | 0.95  0.93  0.85  0.60  0.54 | 0.90 – 1.00  0.90 – 0.96  0.82 – 0.89  0.54 – 0.67  0.21 – 1.40 | 0.050  <0.001  <0.001  <0.001  0.204 | <0.001 |
| Worsening LoC (or death)  65 – 69 years  70 – 79 years  80 – 89 years  90 – 99 years  ≥ 100 years | 0.95  0.95  0.99  1.04  1.37 | 0.86 – 1.05  0.91 – 1.00  0.95 – 1.02  0.97 – 1.11  0.93 – 2.04 | 0.345  0.046  0.479  0.246  0.115 | 0.108 |

**Appendix Table S4:** Event rates with 95% confidence interval (95%CI) for overall mortality (OM); major adverse events (MAEs) and worsening of Level of care (LoC) or death for centenarians with proximal humeral fracture depending on treatment. Event rates were given as 1- survival function S(t) determined by Kaplan-Meier estimates. Only patients who had an observation period of > 21 days were included, as the allocation of treatment was only completed after this time. To analyze the worsening of LoC, only patients treated from 2017 onwards were included. *** censored due data privacy protection.

| Endpoint | Non-operative treatment | Surgical treatment |
| --- | --- | --- |
| OM:  1 year rate  2 years rate  3 years rate  4 years rate  5 years rate  Total no. at risk at 21days after PHF  total no. of events | 51.8% (43.4 – 59.5%)  71.2% (62.8 – 78.0%)  84.0% (75.9 – 89.5%)  94.0% (87.0 – 97.3%)  98.7% (85.9 – 99.9%)  154  132 | 43.8% (26.6 – 59.7%)  71.9% (52.3 – 84.5%)  71.9% (52.3 – 84.5%)  87.5% (68.3 – 95.4%)  93.8% (73.0 – 98.7%)  35  *** |
| MAE:  1 year rate  2 years rate  3 years rate  4 years rate  5 years rate  Total no. at risk at 21days after PHF  total no. of events | 51.7% (43.4 – 59.3%)  69.6% (61.4 – 76.4%)  80.8% (73.0 – 86.6%)  92.2% (84.1 – 96.2%)  97.4% (88.4 – 99.4%)  151  132 | 48.5% (30.4 – 64.4%)  81.9% (61.1 – 92.2%)  81.9% (61.1 – 92.2%)  89.1% (67.7 – 96.7%)  92.8% (69.6 – 98.5%)  33  *** |
| Worsening LoC (or death):  1 year rate  2 years rate  3 years rate  4 years rate  5 years rate  Total no. at risk at 21days after PHF  total no. of events | 68.7% (55.4 – 78.8%)  86.1% (70.6 – 93.8%)  89.6% (72.4 – 96.3%)  89.6% (72.4 – 96.3%)  89.6% (72.4 – 96.3%)  66  54 | 92.2% (37.8 – 99.3%)  92.2% (37.8 – 99.3%)  n.a.  n.a.  n.a.  17  16 |

**Supplementary Table S5:** Results of all regression analysis. CI – confidence interval, LoC – Level of Care, PHF – proximal humeral fracture.

| Variable | HR | 95% CI | p-value |
| --- | --- | --- | --- |
| Overall survival – without interaction | | | |
| Year of PHF (per year) | 1.01 | 1.00 – 1.01 | <0.001 |
| Age at PHF (per year) |  |  |  |
| ≤ 100 years vs (65 – 69 years) | 19.23 | 16.63 – 22.24 | <0.001 |
| (90 – 99 years) vs (65 – 69 years) | 8.57 | 8.13 – 9.03 | <0.001 |
| (80 – 89 years) vs (65 – 69 years) | 3.91 | 3.73 – 4.10 | <0.001 |
| (70 – 79 years) vs (65 – 69 years) | 1.72 | 1.64 – 1.80 | <0.001 |
| Male sex | 1.57 | 1.53 – 1.61 | <0.001 |
| Atrial fibrillation/ flutter | 1.33 | 1.30 – 1.37 | <0.001 |
| Alcohol abuse | 1.60 | 1.53 – 1.67 | <0.001 |
| Atherosclerosis | 1.04 | 1.01 – 1.06 | 0.008 |
| Cancer | 1.10 | 1.08 – 1.13 | <0.001 |
| Congestive heart failure | 1.38 | 1.35 – 1.42 | <0.001 |
| Chronic polyarthritis | 1.04 | 1.00 – 1.08 | 0.069 |
| Chronic kidney disease | 1.30 | 1.27 – 1.33 | <0.001 |
| Dementia | 2.00 | 1.95 – 2.05 | <0.001 |
| Diabetes Mellitus | 1.23 | 1.21 – 1.26 | <0.001 |
| Frozen Shoulder | 0.77 | 0.73 – 0.82 | <0.001 |
| Any anticoagulant | 1.13 | 1.10 – 1.16 | <0.001 |
| Hypertension | 1.04 | 1.01 – 1.08 | 0.019 |
| Coronary heart disease | 1.00 | 0.98 – 1.02 | 0.892 |
| Nicotine abuse | 1.49 | 1.43 – 1.55 | <0.001 |
| Obesity | 0.87 | 0.84 – 0.89 | <0.001 |
| Omarthrosis | 0.92 | 0.86 – 0.98 | 0.0101 |
| Any anti-osteoporotic drugs | 1.13 | 1.09 – 1.17 | <0.001 |
| Osteoporosis | 1.02 | 0.99 – 1.04 | 0.182 |
| Parkinson disease | 1.38 | 1.33 – 1.44 | <0.001 |
| Prev. stroke/other cerebrovascular disease | 1.07 | 1.05 – 1.10 | <0.001 |
| Rotator cuff rupture | 0.75 | 0.70 – 0.81 | <0.001 |
| Outpatient diagnosis | 0.69 | 0.68 – 0.71 | <0.001 |
| Surgical treatment within 21 days (time-dependent) | 0.84 | 0.82 – 0.86 | <0.001 |
| Overall survival – with interaction | | | |
| Year of PHF (per year) | 1.01 | 1.00 – 1.01 | <0.001 |
| Age at PHF (per year) | n.a. | n.a. | <0.001 |
| Male sex | 1.57 | 1.53 – 1.61 | <0.001 |
| Atrial fibrillation/ flutter | 1.33 | 1.30 – 1.37 | <0.001 |
| Alcohol abuse | 1.60 | 1.53 – 1.67 | <0.001 |
| Atherosclerosis | 1.04 | 1.01 – 1.06 | 0.008 |
| Cancer | 1.10 | 1.08 – 1.13 | <0.001 |
| Congestive heart failure | 1.38 | 1.35 – 1.42 | <0.001 |
| Chronic polyarthritis | 1.04 | 1.00 – 1.08 | 0.068 |
| Chronic kidney disease | 1.30 | 1.27 – 1.33 | <0.001 |
| Dementia | 2.00 | 1.95 – 2.05 | <0.001 |
| Diabetes Mellitus | 1.23 | 1.21 – 1.26 | <0.001 |
| Frozen Shoulder | 0.77 | 0.73 – 0.82 | <0.001 |
| Any anticoagulant | 1.13 | 1.10 – 1.16 | <0.001 |
| Hypertension | 1.04 | 1.01 – 1.08 | 0.020 |
| Coronary heart disease | 1.00 | 0.98 – 1.02 | 0.888 |
| Nicotine abuse | 1.49 | 1.43 – 1.55 | <0.001 |
| Obesity | 0.87 | 0.84 – 0.89 | <0.001 |
| Omarthrosis | 0.92 | 0.86 – 0.98 | 0.010 |
| Any anti-osteoporotic drugs | 1.13 | 1.09 – 1.17 | <0.001 |
| Osteoporosis | 1.02 | 0.99 – 1.04 | 0.184 |
| Parkinson disease | 1.38 | 1.33 – 1.44 | <0.001 |
| Prev. stroke/other cerebrovascular disease | 1.07 | 1.05 – 1.10 | <0.001 |
| Rotator cuff rupture | 0.75 | 0.70 – 0.81 | <0.001 |
| Outpatient diagnosis | 0.69 | 0.68 – 0.71 | <0.001 |
| Surgical treatment within 21 days (time-dependent) | n.a. | n.a. | <0.001 |
| Age*Surgical treatment within 21 days (time-dependent) | n.a. | n.a. | 0.835 |
| Major adverse events – without interaction | | | |
| Year of PHF (per year) | 0.97 | 0.96 – 0.97 | <0.001 |
| Age at PHF (per year) |  |  |  |
| ≤ 100 years vs (65 – 69 years) | 9.17 | 7.96 – 10.57 | <0.001 |
| (90 – 99 years) vs (65 – 69 years) | 4.98 | 4.77 – 5.21 | <0.001 |
| (80 – 89 years) vs (65 – 69 years) | 2.74 | 2.63 – 2.84 | <0.001 |
| (70 – 79 years) vs (65 – 69 years) | 1.50 | 1.45 – 1.56 | <0.001 |
| Male sex | 1.51 | 1.47 – 1.54 | <0.001 |
| Atrial fibrillation/ flutter | 1.30 | 1.27 – 1.33 | <0.001 |
| Alcohol abuse | 1.62 | 1.56 – 1.69 | <0.001 |
| Atherosclerosis | 1.04 | 1.02 – 1.07 | 0.001 |
| Cancer | 1.08 | 1.06 – 1.10 | <0.001 |
| Congestive heart failure | 1.34 | 1.31 – 1.37 | <0.001 |
| Chronic polyarthritis | 1.08 | 1.04 – 1.12 | <0.001 |
| Chronic kidney disease | 1.37 | 1.34 – 1.40 | <0.001 |
| Dementia | 1.73 | 1.69 – 1.77 | <0.001 |
| Diabetes Mellitus | 1.24 | 1.22 – 1.26 | <0.001 |
| Frozen Shoulder | 0.84 | 0.80 – 0.88 | <0.001 |
| Any anticoagulant | 1.14 | 1.11 – 1.16 | <0.001 |
| Hypertension | 1.13 | 1.10 – 1.17 | <0.001 |
| Coronary heart disease | 1.03 | 1.00 – 1.05 | 0.018 |
| Nicotine abuse | 1.42 | 1.36 – 1.47 | <0.001 |
| Obesity | 0.94 | 0.92 – 0.96 | <0.001 |
| Omarthrosis | 0.95 | 0.09 – 1.00 | 0.068 |
| Any anti-osteoporotic drugs | 1.10 | 1.07 – 1.14 | <0.001 |
| Osteoporosis | 1.01 | 0.99 – 1.03 | 0.318 |
| Parkinson disease | 1.32 | 1.27 – 1.37 | <0.001 |
| Prev. stroke/other cerebrovascular disease | 1.12 | 1.10 – 1.14 | <0.001 |
| Rotator cuff rupture | 0.82 | 0.77 – 0.88 | <0.001 |
| Outpatient diagnosis | 0.65 | 0.64 – 0.67 | <0.001 |
| Surgical treatment within 21 days (time-dependent) | 0.72 | 0.71 – 0.74 | <0.001 |
| Major adverse events – with interaction |  |  |  |
| Year of PHF (per year) | 0.97 | 0.96 – 0.97 | <0.001 |
| Age at PHF (per year) | n.a. | n.a. | <0.001 |
| Male sex | 1.51 | 1.47 – 1.54 | <0.001 |
| Atrial fibrillation/ flutter | 1.30 | 1.27 – 1.33 | <0.001 |
| Alcohol abuse | 1.62 | 1.56 – 1.69 | <0.001 |
| Atherosclerosis | 1.04 | 1.02 – 1.07 | 0.001 |
| Cancer | 1.08 | 1.06 – 1.10 | <0.001 |
| Congestive heart failure | 1.34 | 1.31 – 1.37 | <0.001 |
| Chronic polyarthritis | 1.08 | 1.04 – 1.12 | <0.001 |
| Chronic kidney disease | 1.37 | 1.34 – 1.40 | <0.001 |
| Dementia | 1.73 | 1.69 – 1.77 | <0.001 |
| Diabetes Mellitus | 1.24 | 1.22 – 1.26 | <0.001 |
| Frozen Shoulder | 0.84 | 0.80 – 0.88 | <0.001 |
| Any anticoagulant | 1.14 | 1.11 – 1.16 | <0.001 |
| Hypertension | 1.13 | 1.10 – 1.17 | <0.001 |
| Coronary heart disease | 1.03 | 1.00 – 1.05 | 0.018 |
| Nicotine abuse | 1.42 | 1.36 – 1.47 | <0.001 |
| Obesity | 0.94 | 0.92 – 0.96 | <0.001 |
| Omarthrosis | 0.95 | 0.90 – 1.00 | 0.065 |
| Any anti-osteoporotic drugs | 1.10 | 1.07 – 1.14 | <0.001 |
| Osteoporosis | 1.01 | 0.99 – 1.03 | 0.326 |
| Parkinson disease | 1.32 | 1.27 – 1.37 | <0.001 |
| Prev. stroke/other cerebrovascular disease | 1.12 | 1.10 – 1.14 | <0.001 |
| Rotator cuff rupture | 0.82 | 0.77 – 0.87 | <0.001 |
| Outpatient diagnosis | 0.65 | 0.64 – 0.67 | <0.001 |
| Surgical treatment within 21 days (time-dependent) | n.a. | n.a. | <0.001 |
| Age*Surgical treatment within 21 days (time-dependent) | n.a. | n.a. | 0.059 |
| Thromboembolic events – without interaction | | | |
| Year of PHF (per year) | 0.96 | 0.96 – 0.96 | <0.001 |
| Age at PHF (per year) |  |  |  |
| ≤ 100 years vs (65 – 69 years) | 11.75 | 10.18 – 13.56 | <0.001 |
| (90 – 99 years) vs (65 – 69 years) | 5.98 | 5.71 – 6.27 | <0.001 |
| (80 – 89 years) vs (65 – 69 years) | 3.07 | 2.95 – 3.21 | <0.001 |
| (70 – 79 years) vs (65 – 69 years) | 1.58 | 1.52 – 1.65 | <0.001 |
| Male sex | 1.48 | 1.45 – 1.52 | <0.001 |
| Atrial fibrillation/ flutter | 1.26 | 1.23 – 1.29 | <0.001 |
| Alcohol abuse | 1.53 | 1.47 – 1.60 | <0.001 |
| Atherosclerosis | 1.04 | 1.02 – 1.07 | 0.001 |
| Cancer | 1.10 | 1.08 – 1.12 | <0.001 |
| Congestive heart failure | 1.33 | 1.30 – 1.36 | <0.001 |
| Chronic polyarthritis | 1.06 | 1.02 – 1.10 | 0.007 |
| Chronic kidney disease | 1.27 | 1.25 – 1.30 | <0.001 |
| Dementia | 1.85 | 1.81 – 1.90 | <0.001 |
| Diabetes Mellitus | 1.20 | 1.18 – 1.23 | <0.001 |
| Frozen Shoulder | 0.82 | 0.78 – 0.86 | <0.001 |
| Any anticoagulant | 1.13 | 1.10 – 1.15 | <0.001 |
| Hypertension | 1.07 | 1.04 – 1.11 | <0.001 |
| Coronary heart disease | 1.00 | 0.98 – 1.02 | 0.761 |
| Nicotine abuse | 1.41 | 1.35 – 1.47 | <0.001 |
| Obesity | 0.90 | 0.88 – 0.92 | <0.001 |
| Omarthrosis | 0.95 | 0.89 – 1.01 | 0.086 |
| Any anti-osteoporotic drugs | 1.11 | 1.08 – 1.14 | <0.001 |
| Osteoporosis | 1.02 | 1.00 – 1.04 | 0.0489 |
| Parkinson disease | 1.32 | 1.27 – 1.37 | <0.001 |
| Prev. stroke/other cerebrovascular disease | 1.12 | 1.09 – 1.14 | <0.001 |
| Rotator cuff rupture | 0.80 | 0.75 – 0.85 | <0.001 |
| Outpatient diagnosis | 0.66 | 0.65 – 0.68 | <0.001 |
| Surgical treatment within 21 days (time-dependent) | 0.70 | 0.69 – 0.72 | <0.001 |
| Thromboembolic events – with interaction | | | |
| Year of PHF (per year) | 0.96 | 0.96 – 0.96 | <0.001 |
| Age at PHF (per year) | n.a. | n.a. | <0.001 |
| Male sex | 1.48 | 1.45 – 1.52 | <0.001 |
| Atrial fibrillation/ flutter | 1.26 | 1.23 – 1.29 | <0.001 |
| Alcohol abuse | 1.53 | 1.47 – 1.60 | <0.001 |
| Atherosclerosis | 1.04 | 1.02 – 1.07 | 0.001 |
| Cancer | 1.10 | 1.08 – 1.12 | <0.001 |
| Congestive heart failure | 1.33 | 1.30 – 1.36 | <0.001 |
| Chronic polyarthritis | 1.06 | 1.02 – 1.10 | 0.007 |
| Chronic kidney disease | 1.27 | 1.24 – 1.30 | <0.001 |
| Dementia | 1.85 | 1.81 – 1.90 | <0.001 |
| Diabetes Mellitus | 1.20 | 1.18 – 1.23 | <0.001 |
| Frozen Shoulder | 0.82 | 0.78 – 0.86 | <0.001 |
| Any anticoagulant | 1.13 | 1.10 – 1.15 | <0.001 |
| Hypertension | 1.07 | 1.04 – 1.11 | <0.001 |
| Coronary heart disease | 1.00 | 0.98 – 1.02 | 0.756 |
| Nicotine abuse | 1.41 | 1.35 – 1.47 | <0.001 |
| Obesity | 0.90 | 0.88 – 0.92 | <0.001 |
| Omarthrosis | 0.95 | 0.89 – 1.01 | 0.083 |
| Any anti-osteoporotic drugs | 1.11 | 1.08 – 1.14 | <0.001 |
| Osteoporosis | 1.02 | 1.00 – 1.04 | 0.049 |
| Parkinson disease | 1.32 | 1.27 – 1.37 | <0.001 |
| Prev. stroke/other cerebrovascular disease | 1.12 | 1.09 – 1.14 | <0.001 |
| Rotator cuff rupture | 0.80 | 0.75 – 0.85 | <0.001 |
| Outpatient diagnosis | 0.66 | 0.65 – 0.68 | <0.001 |
| Surgical treatment within 21 days (time-dependent) | n.a. | n.a. | <0.001 |
| Age*Surgical treatment within 21 days (time-dependent) | n.a. | n.a. | 0.200 |
| Worsening LoC (or death) – without interaction | | | |
| Year of PHF (per year) | 1.04 | 1.03 – 1.05 | <0.001 |
| Age at PHF (per year) |  |  |  |
| ≤ 100 years vs (65 – 69 years) | 10.80 | 8.87 – 13.14 | <0.001 |
| (90 – 99 years) vs (65 – 69 years) | 7.15 | 6.71 – 7.62 | <0.001 |
| (80 – 89 years) vs (65 – 69 years) | 4.37 | 4.13 – 4.63 | <0.001 |
| (70 – 79 years) vs (65 – 69 years) | 2.06 | 1.95 – 2.18 | <0.001 |
| Male sex | 1.17 | 1.13 – 1.20 | <0.001 |
| Atrial fibrillation/ flutter | 1.14 | 1.11 – 1.18 | <0.001 |
| Alcohol abuse | 1.57 | 1.49 – 1.66 | <0.001 |
| Atherosclerosis | 1.02 | 0.99 – 1.05 | 0.310 |
| Cancer | 1.05 | 1.02 – 1.07 | 0.001 |
| Congestive heart failure | 1.19 | 1.16 – 1.23 | <0.001 |
| Chronic polyarthritis | 1.08 | 1.03 – 1.13 | 0.003 |
| Chronic kidney disease | 1.13 | 1.10 – 1.16 | <0.001 |
| Dementia | 1.47 | 1.43 – 1.52 | <0.001 |
| Diabetes Mellitus | 1.18 | 1.15 – 1.21 | <0.001 |
| Frozen Shoulder | 0.90 | 0.84 – 0.96 | 0.001 |
| Any anticoagulant | 1.03 | 1.00 – 1.06 | 0.064 |
| Hypertension | 1.12 | 1.07 – 1.16 | <0.001 |
| Coronary heart disease | 1.03 | 1.00 – 1.06 | 0.028 |
| Nicotine abuse | 1.23 | 1.17 – 1.29 | <0.001 |
| Obesity | 1.07 | 1.04 – 1.11 | <0.001 |
| Omarthrosis | 0.94 | 0.88 – 1.01 | 0.083 |
| Any anti-osteoporotic drugs | 1.08 | 1.04 – 1.11 | <0.001 |
| Osteoporosis | 1.06 | 1.03 – 1.09 | <0.001 |
| Parkinson disease | 1.36 | 1.29 – 1.43 | <0.001 |
| Prev. stroke/other cerebrovascular disease | 1.07 | 1.05 – 1.10 | <0.001 |
| Rotator cuff rupture | 0.86 | 0.79 – 0.93 | 0.000 |
| Outpatient diagnosis | 0.62 | 0.60 – 0.63 | <0.001 |
| Surgical treatment within 21 days (time-dependent) | 0.99 | 0.96 – 1.01 | 0.259 |
| Worsening LoC (or death) – with interaction | | | |
| Year of PHF (per year) | 1.04 | 1.03 – 1.05 | <0.001 |
| Age at PHF (per year) | n.a. | n.a. | <0.001 |
| Male sex | 1.16 | 1.13 – 1.20 | <0.001 |
| Atrial fibrillation/ flutter | 1.14 | 1.11 – 1.18 | <0.001 |
| Alcohol abuse | 1.57 | 1.49 – 1.66 | <0.001 |
| Atherosclerosis | 1.02 | 0.98 – 1.05 | 0.333 |
| Cancer | 1.05 | 1.02 – 1.07 | 0.001 |
| Congestive heart failure | 1.19 | 1.16 – 1.23 | <0.001 |
| Chronic polyarthritis | 1.08 | 1.02 – 1.13 | 0.004 |
| Chronic kidney disease | 1.13 | 1.10 – 1.16 | <0.001 |
| Dementia | 1.47 | 1.42 – 1.52 | <0.001 |
| Diabetes Mellitus | 1.18 | 1.15 – 1.21 | <0.001 |
| Frozen Shoulder | 0.90 | 0.84 – 0.96 | 0.001 |
| Any anticoagulant | 1.03 | 1.00 – 1.06 | 0.075 |
| Hypertension | 1.12 | 1.07 – 1.16 | <0.001 |
| Coronary heart disease | 1.03 | 1.00 – 1.06 | 0.035 |
| Nicotine abuse | 1.23 | 1.17 – 1.29 | <0.001 |
| Obesity | 1.07 | 1.04 – 1.11 | <0.001 |
| Omarthrosis | 0.94 | 0.88 – 1.01 | 0.087 |
| Any anti-osteoporotic drugs | 1.08 | 1.03 – 1.12 | <0.001 |
| Osteoporosis | 1.06 | 1.03 – 1.09 | <0.001 |
| Parkinson disease | 1.36 | 1.29 – 1.44 | <0.001 |
| Prev. stroke/other cerebrovascular disease | 1.07 | 1.04 – 1.10 | <0.001 |
| Rotator cuff rupture | 0.86 | 0.79 – 0.93 | <0.001 |
| Outpatient diagnosis | 0.61 | 0.60 – 0.63 | <0.001 |
| Surgical treatment within 21 days (time-dependent) | n.a. | n.a. | 0.345 |
| Age*Surgical treatment within 21 days (time-dependent) | n.a. | n.a. | 0.098 |
| Surgical or injured-related complication – without interaction | | | |
| Year of PHF (per year) | 0.98 | 0.97 – 0.98 | <0.001 |
| Age at PHF (per year) |  |  |  |
| ≤ 100 years vs (65 – 69 years) | 0.20 | 0.09 – 0.49 | <0.001 |
| (90 – 99 years) vs (65 – 69 years) | 0.41 | 0.37 – 0.45 | <0.001 |
| (80 – 89 years) vs (65 – 69 years) | 0.65 | 0.61 – 0.70 | <0.001 |
| (70 – 79 years) vs (65 – 69 years) | 0.89 | 0.84 – 0.94 | <0.001 |
| Male sex | 1.07 | 1.01 – 1.13 | 0.024 |
| Atrial fibrillation/ flutter | 0.95 | 0.90 – 1.01 | 0.132 |
| Alcohol abuse | 1.46 | 1.35 – 1.58 | <0.001 |
| Atherosclerosis | 1.02 | 0.96 – 1.08 | 0.534 |
| Cancer | 1.05 | 1.00 – 1.10 | 0.048 |
| Congestive heart failure | 1.00 | 0.94 – 1.05 | 0.902 |
| Chronic polyarthritis | 1.10 | 1.02 – 1.19 | 0.013 |
| Chronic kidney disease | 1.05 | 1.00 – 1.11 | 0.061 |
| Dementia | 0.76 | 0.71 – 0.82 | <0.001 |
| Diabetes Mellitus | 0.99 | 0.95 – 1.04 | 0.713 |
| Frozen Shoulder | 1.21 | 1.10 – 1.32 | <0.001 |
| Any anticoagulant | 1.04 | 0.98 – 1.09 | 0.198 |
| Hypertension | 1.10 | 1.04 – 1.17 | 0.001 |
| Coronary heart disease | 0.99 | 0.94 – 1.04 | 0.718 |
| Nicotine abuse | 1.06 | 0.98 – 1.15 | 0.128 |
| Obesity | 1.26 | 1.20 – 1.32 | <0.001 |
| Omarthrosis | 1.13 | 1.01 – 1.27 | 0.038 |
| Any anti-osteoporotic drugs | 1.04 | 0.97 – 1.11 | 0.254 |
| Osteoporosis | 1.19 | 1.13 – 1.24 | <0.001 |
| Parkinson disease | 1.20 | 1.08 – 1.33 | 0.001 |
| Prev. stroke/other cerebrovascular disease | 0.93 | 0.88 – 0.98 | 0.004 |
| Rotator cuff rupture | 1.45 | 1.31 – 1.62 | <0.001 |
| Outpatient diagnosis | 0.76 | 0.73 – 0.80 | <0.001 |
| Surgical treatment within 21 days (time-dependent) | 1.28 | 1.22 – 1.38 | 0.001 |
| Surgical or injured-related complication – with interaction | | | |
| Year of PHF (per year) | 0.98 | 0.97 – 0.98 | <0.001 |
| Age at PHF (per year) | n.a. | n.a. | <0.001 |
| Male sex | 1.07 | 1.01 – 1.13 | 0.025 |
| Atrial fibrillation/ flutter | 0.95 | 0.90 – 1.01 | 0.128 |
| Alcohol abuse | 1.46 | 1.35 – 1.58 | <0.001 |
| Atherosclerosis | 1.02 | 0.96 – 1.08 | 0.546 |
| Cancer | 1.05 | 1.00 – 1.10 | 0.049 |
| Congestive heart failure | 1.00 | 0.94 – 1.05 | 0.908 |
| Chronic polyarthritis | 1.10 | 1.02 – 1.19 | 0.013 |
| Chronic kidney disease | 1.05 | 1.00 – 1.11 | 0.059 |
| Dementia | 0.76 | 0.71 – 0.82 | <0.001 |
| Diabetes Mellitus | 0.99 | 0.95 – 1.04 | 0.705 |
| Frozen Shoulder | 1.21 | 1.10 – 1.32 | <0.001 |
| Any anticoagulant | 1.04 | 0.98 – 1.09 | 0.200 |
| Hypertension | 1.10 | 1.04 – 1.17 | 0.001 |
| Coronary heart disease | 0.99 | 0.94 – 1.04 | 0.712 |
| Nicotine abuse | 1.06 | 0.98 – 1.15 | 0.133 |
| Obesity | 1.26 | 1.20 – 1.32 | <0.001 |
| Omarthrosis | 1.13 | 1.01 – 1.27 | 0.039 |
| Any anti-osteoporotic drugs | 1.04 | 0.97 – 1.11 | 0.258 |
| Osteoporosis | 1.19 | 1.13 – 1.24 | <0.001 |
| Parkinson disease | 1.20 | 1.08 – 1.33 | 0.001 |
| Prev. stroke/other cerebrovascular disease | 0.93 | 0.88 – 0.98 | 0.004 |
| Rotator cuff rupture | 1.46 | 1.31 – 1.62 | <0.001 |
| Outpatient diagnosis | 0.76 | 0.73 – 0.80 | <0.001 |
| Surgical treatment within 21 days (time-dependent) | n.a. | n.a. | <0.001 |
| Age*Surgical treatment within 21 days (time-dependent) | n.a. | n.a. | 0.047 |
| Minor outpatient complications – without interaction | | | |
| Year of PHF (per year) | 1.00 | 1.00 – 1.00 | 0.970 |
| Age at PHF (per year) |  |  |  |
| ≤ 100 years vs (65 – 69 years) | 0.24 | 0.16 – 0.38 | <0.001 |
| (90 – 99 years) vs (65 – 69 years) | 0.37 | 0.35 – 0.40 | <0.001 |
| (80 – 89 years) vs (65 – 69 years) | 0.68 | 0.66 – 0.71 | <0.001 |
| (70 – 79 years) vs (65 – 69 years) | 0.94 | 0.91 – 0.97 | <0.001 |
| Male sex | 0.96 | 0.93 – 0.99 | 0.014 |
| Atrial fibrillation/ flutter | 0.87 | 0.84 – 0.90 | <0.001 |
| Alcohol abuse | 0.96 | 0.91 – 1.01 | 0.114 |
| Atherosclerosis | 1.05 | 1.02 – 1.09 | 0.001 |
| Cancer | 1.04 | 1.01 – 1.07 | 0.003 |
| Congestive heart failure | 0.91 | 0.88 – 0.94 | <0.001 |
| Chronic polyarthritis | 1.09 | 1.05 – 1.14 | <0.001 |
| Chronic kidney disease | 0.96 | 0.93 – 0.99 | 0.012 |
| Dementia | 0.59 | 0.56 – 0.61 | <0.001 |
| Diabetes Mellitus | 0.93 | 0.91 – 0.95 | <0.001 |
| Frozen Shoulder | 2.95 | 2.82 – 3.08 | <0.001 |
| Any anticoagulant | 0.95 | 0.92 – 0.98 | <0.001 |
| Hypertension | 1.08 | 1.05 – 1.12 | <0.001 |
| Coronary heart disease | 1.06 | 1.03 – 1.09 | <0.001 |
| Nicotine abuse | 0.93 | 0.89 – 0.98 | 0.003 |
| Obesity | 1.20 | 1.17 – 1.24 | <0.001 |
| Omarthrosis | 2.24 | 2.12 – 2.37 | <0.001 |
| Any anti-osteoporotic drugs | 1.05 | 1.01 – 1.09 | 0.014 |
| Osteoporosis | 1.14 | 1.11 – 1.17 | <0.001 |
| Parkinson disease | 0.90 | 0.84 – 0.96 | 0.001 |
| Prev. stroke/other cerebrovascular disease | 1.06 | 1.03 – 1.08 | <0.001 |
| Rotator cuff rupture | 3.25 | 3.07 – 3.44 | <0.001 |
| Outpatient diagnosis | 1.12 | 1.09 – 1.15 | <0.001 |
| Surgical treatment within 21 days (time-dependent) | 0.89 | 0.87 – 0.91 | <0.001 |
| Minor outpatient complications – with interaction | | | |
| Year of PHF (per year) | 1.00 | 1.00 – 1.00 | 0.951 |
| Age at PHF (per year) | n.a. | n.a. | <0.001 |
| Male sex | 0.96 | 0.93 – 0.99 | 0.016 |
| Atrial fibrillation/ flutter | 0.87 | 0.84 – 0.90 | <0.001 |
| Alcohol abuse | 0.96 | 0.91 – 1.01 | 0.099 |
| Atherosclerosis | 1.05 | 1.02 – 1.09 | 0.001 |
| Cancer | 1.04 | 1.01 – 1.07 | 0.003 |
| Congestive heart failure | 0.91 | 0.88 – 0.94 | <0.001 |
| Chronic polyarthritis | 1.09 | 1.05 – 1.14 | <0.001 |
| Chronic kidney disease | 0.96 | 0.93 – 0.99 | 0.015 |
| Dementia | 0.59 | 0.56 – 0.61 | <0.001 |
| Diabetes Mellitus | 0.93 | 0.91 – 0.95 | <0.001 |
| Frozen Shoulder | 2.95 | 2.82 – 3.08 | <0.001 |
| Any anticoagulant | 0.95 | 0.92 – 0.97 | <0.001 |
| Hypertension | 1.08 | 1.05 – 1.12 | <0.001 |
| Coronary heart disease | 1.06 | 1.03 – 1.09 | <0.001 |
| Nicotine abuse | 0.93 | 0.89 – 0.98 | 0.003 |
| Obesity | 1.20 | 1.17 – 1.23 | <0.001 |
| Omarthrosis | 2.25 | 2.12 – 2.38 | <0.001 |
| Any anti-osteoporotic drugs | 1.05 | 1.01 – 1.09 | 0.016 |
| Osteoporosis | 1.14 | 1.11 – 1.17 | <0.001 |
| Parkinson disease | 0.90 | 0.84 – 0.96 | 0.001 |
| Prev. stroke/other cerebrovascular disease | 1.06 | 1.03 – 1.08 | <0.001 |
| Rotator cuff rupture | 3.27 | 3.09 – 3.46 | <0.001 |
| Outpatient diagnosis | 1.13 | 1.10 – 1.15 | <0.001 |
| Surgical treatment within 21 days (time-dependent) | n.a. | n.a. | 0.050 |
| Age*Surgical treatment within 21 days (time-dependent) | n.a. | n.a. | <0.001 |
| Secondary osteoporosis associated fractures | | | |
| Surgical treatment within 21 days (time-dependent) | 0.65 | 0.63 – 0.68 | <0.001 |
| Year of PHF (per year) | 0.97 | 0.97 – 0.98 | <0.001 |
| Age at PHF (per year) |  |  |  |
| ≤ 100 years vs (65 – 69 years) | 1.35 | 0.94 – 1.93 | 0.104 |
| (90 – 99 years) vs (65 – 69 years) | 1.70 | 1.58 – 1.83 | <0.001 |
| (80 – 89 years) vs (65 – 69 years) | 2.04 | 1.93 – 2.16 | <0.001 |
| (70 – 79 years) vs (65 – 69 years) | 1.58 | 1.50 – 1.66 | <0.001 |
| Male sex | 0.79 | 0.76 – 0.83 | <0.001 |
| Atrial fibrillation/ flutter | 1.02 | 0.98 – 1.06 | 0.369 |
| Alcohol abuse | 1.31 | 1.22 – 1.40 | <0.001 |
| Atherosclerosis | 1.06 | 1.02 – 1.10 | 0.006 |
| Cancer | 1.01 | 0.97 – 1.04 | 0.737 |
| Congestive heart failure | 0.94 | 0.91 – 0.98 | 0.002 |
| Chronic polyarthritis | 1.12 | 1.06 – 1.18 | <0.001 |
| Chronic kidney disease | 0.93 | 0.90 – 0.97 | <0.001 |
| Dementia | 0.92 | 0.88 – 0.97 | 0.001 |
| Diabetes Mellitus | 0.98 | 0.95 – 1.02 | 0.273 |
| Frozen Shoulder | 0.97 | 0.91 – 1.04 | 0.453 |
| Any anticoagulant | 1.03 | 0.99 – 1.06 | 0.200 |
| Hypertension | 1.01 | 0.97 – 1.05 | 0.728 |
| Coronary heart disease | 1.00 | 0.96 – 1.03 | 0.918 |
| Nicotine abuse | 1.17 | 1.10 – 1.25 | <0.001 |
| Obesity | 0.85 | 0.82 – 0.89 | <0.001 |
| Omarthrosis | 1.00 | 0.91 – 1.09 | 0.915 |
| Parkinson disease | 1.23 | 1.14 – 1.31 | <0.001 |
| Prev. stroke/other cerebrovascular disease | 1.05 | 1.01 – 1.08 | 0.011 |
| Rotator cuff rupture | 0.97 | 0.89 – 1.06 | 0.512 |
| Outpatient diagnosis | 0.78 | 0.76 – 0.81 | <0.001 |
| Osteoporosis incl. follow-up (time-dependent) | 1.79 | 1.73 – 1.85 | <0.001 |
| Any anti-osteoporotic drugs incl. follow-up (time-dependent) | 0.56 | 0.53 – 0.58 | <0.001 |
| Secondary osteoporosis associated fractures (incl. ICD M80) | | | |
| Surgical treatment within 21 days (time-dependent) | 1.06 | 1.03 – 1.08 | <0.001 |
| Year of PHF (per year) | 0.99 | 0.99 – 0.99 | <0.001 |
| Age at PHF (per year) |  |  |  |
| ≤ 100 years vs (65 – 69 years) | 1.86 | 1.38 – 2.50 | <0.001 |
| (90 – 99 years) vs (65 – 69 years) | 1.89 | 1.78 – 2.00 | <0.001 |
| (80 – 89 years) vs (65 – 69 years) | 1.78 | 1.70 – 1.87 | <0.001 |
| (70 – 79 years) vs (65 – 69 years) | 1.41 | 1.35 – 1.47 | <0.001 |
| Male sex | 0.94 | 0.91 – 0.98 | 0.004 |
| Atrial fibrillation/ flutter | 1.06 | 1.02 – 1.10 | 0.002 |
| Alcohol abuse | 1.33 | 1.26 – 1.41 | <0.001 |
| Atherosclerosis | 1.04 | 1.01 – 1.08 | 0.010 |
| Cancer | 1.00 | 0.97 – 1.03 | 0.876 |
| Congestive heart failure | 1.00 | 0.96 – 1.03 | 0.751 |
| Chronic polyarthritis | 1.07 | 1.02 – 1.12 | 0.003 |
| Chronic kidney disease | 0.98 | 0.95 – 1.01 | 0.123 |
| Dementia | 1.05 | 1.01 – 1.09 | 0.014 |
| Diabetes Mellitus | 0.98 | 0.95 – 1.01 | 0.134 |
| Frozen Shoulder | 0.93 | 0.88 – 0.98 | 0.012 |
| Any anticoagulant | 1.05 | 1.02 – 1.09 | 0.001 |
| Hypertension | 0.98 | 0.95 – 1.02 | 0.292 |
| Coronary heart disease | 1.00 | 0.97 – 1.03 | 0.870 |
| Nicotine abuse | 1.17 | 1.11 – 1.23 | <0.001 |
| Obesity | 0.86 | 0.83 – 0.89 | <0.001 |
| Omarthrosis | 0.92 | 0.85 – 0.99 | 0.024 |
| Parkinson disease | 1.21 | 1.14 – 1.28 | <0.001 |
| Prev. stroke/other cerebrovascular disease | 1.02 | 0.99 – 1.05 | 0.269 |
| Rotator cuff rupture | 0.88 | 0.82 – 0.95 | 0.002 |
| Outpatient diagnosis | 0.94 | 0.91 – 0.97 | <0.001 |
| Osteoporosis incl. follow-up (time-dependent) | 4.46 | 4.33 – 4.59 | <0.001 |
| Any anti-osteoporotic drugs incl. follow-up (time-dependent) | 1.41 | 1.38 – 1.45 | <0.001 |
